# Tissue-specific transcriptional imprinting and heterogeneity in human innate lymphoid cells revealed by full-length single-cell RNA-sequencing

**DOI:** 10.1038/s41422-020-00445-x

**Published:** 2021-01-08

**Authors:** Luca Mazzurana, Paulo Czarnewski, Viktor Jonsson, Leif Wigge, Markus Ringnér, Teresa C. Williams, Avinash Ravindran, Åsa K. Björklund, Jesper Säfholm, Gunnar Nilsson, Sven-Erik Dahlén, Ann-Charlotte Orre, Mamdoh Al-Ameri, Charlotte Höög, Charlotte Hedin, Sylwester Szczegielniak, Sven Almer, Jenny Mjösberg

**Affiliations:** 1 Department of Medicine, Center for Infectious Medicine, Karolinska Institutet, Stockholm, Sweden; 2 Science for Life Laboratory, Department of Biochemistry and Biophysics, National Bioinformatics Infrastructure Sweden, Stockholm University, Solna, Sweden; 3 Science for Life Laboratory, Department of Biology and Biological Engineering, National Bioinformatics Infrastructure Sweden, Chalmers University of Technology, Gothenburg, Sweden; 4 Science for Life Laboratory, Department of Biology, National Bioinformatics Infrastructure Sweden, Lund University, Lund, Sweden; 5 Immunology and Allergy Unit, Department of Medicine Solna, Department of Clinical Immunology and Transfusion Medicine, Karolinska Institutet, Karolinska University Hospital, Stockholm, Sweden; 6 Science for Life Laboratory, Department of Cell and Molecular Biology, National Bioinformatics Infrastructure Sweden, Uppsala University, Uppsala, Sweden; 7 Institutet of Environmental Medicine, Center for Allergy Research, Karolinska Institutet, Stockholm, Sweden; 8 Division of Thoracic Surgery, Karolinska University Hospital, Stockholm, Sweden; 9 Department of Molecular Medicine and Surgery, Karolinska Institutet, Stockholm, Sweden; 10 Department of Medicine Solna, Karolinska Institutet, Stockholm, Sweden; 11 Division of Gastroenterology, Medical Unit Gastroenterology, Dermatovenereology and Rheumatology, Karolinska University Hospital, Stockholm, Sweden

**Keywords:** Innate immunity, Bioinformatics

## Abstract

The impact of the microenvironment on innate lymphoid cell (ILC)-mediated immunity in humans remains largely unknown. Here we used full-length Smart-seq2 single-cell RNA-sequencing to unravel tissue-specific transcriptional profiles and heterogeneity of CD127^+^ ILCs across four human tissues. Correlation analysis identified gene modules characterizing the migratory properties of tonsil and blood ILCs, and signatures of tissue-residency, activation and modified metabolism in colon and lung ILCs. Trajectory analysis revealed potential differentiation pathways from circulating and tissue-resident naïve ILCs to a spectrum of mature ILC subsets. In the lung we identified both CRTH2^+^ and CRTH2^−^ ILC2 with lung-specific signatures, which could be recapitulated by alarmin-exposure of circulating ILC2. Finally, we describe unique TCR-V(D)J-rearrangement patterns of blood ILC1-like cells, revealing a subset of potentially immature ILCs with TCR-δ rearrangement. Our study provides a useful resource for in-depth understanding of ILC-mediated immunity in humans, with implications for disease.

## Introduction

Innate lymphoid cells (ILCs) are a recent addition to innate immunity, playing an important role in clearing intra- and extracellular pathogens, regulating adaptive immunity, tissue repair and contributing to inflammation.^1^ Their defining feature is a lack of rearranged antigen-specific receptors, which distinguishes them from T cells. However, in other ways these cells closely mirror the function of their adaptive counterparts, being an essential source of cytokines related to type 1, 2 and 17 immune responses.^1^ Currently, ILCs are classified into five subsets: ILC1, ILC2, ILC3, Natural killer (NK) cells and lymphoid tissue inducer (LTi) cells. ILC1 produce interferon-gamma (IFN-γ) and are dependent on the T-box transcription factor (TF) TBX21 (Tbet) for their development and function. NK cells share these features with ILC1, but they additionally express the TF Eomesodermin homolog (Eomes) and have the ability to produce cytolytic and cytotoxic molecules.^1^ ILC2s produce IL-13 and IL-5, and are dependent on GATA-3.^1^ Both LTi cells and ILC3 can produce IL-17 and IL-22 cytokines, and are dependent on the nuclear receptor ROR-γt.^1^ Transcriptional analysis of ILCs obtained from different human and mouse tissues has revealed tissue-specific transcriptional imprinting.^2–4^ However, these studies relied on biased definition of ILC populations sorted in bulk, masking potential heterogeneity and misdefinition of ILC populations across tissues. Recent developments in single-cell technologies on the transcriptional and protein level have rapidly advanced our understanding of the heterogeneity of the human immune system.^5^ While protein analyses still suffer from limitations, e.g., in terms of cell number requirements, single-cell RNA-sequencing (scRNA-seq) enables an unbiased, transcriptome-wide analysis of even small numbers of cells. So far, scRNA-seq of specific subsets of ILCs (NK cells,^6^ ILC2^7^) or in specific tissues^8,9^ has been performed in mice. Similarly, in humans, scRNA-seq has been restricted to one subset (NK cells^6^) or one tissue (ileum^10,11^ and tonsil^12^) at a time, yielding limited understanding of tissue-specific imprinting of ILCs.

In this study, we analyzed human ILCs through full-length scRNA-seq, combining data from four different tissues: blood, tonsil, lung and colon. Comparison between tissues revealed tissue-specific transcriptional imprinting, and gave us the possibility to, for the first time in humans, compare scRNA-seq profiles of ILCs in the circulation, secondary lymphoid organs (SLOs) and mucosal tissues. Our data, which is provided as a publically available resource, reveal previously unknown functions of human ILCs and extend our current understanding of human ILCs biology.

## Results

### ScRNA-seq reveals subset- and tissue-specific transcriptional imprinting

**Fig. 1:**
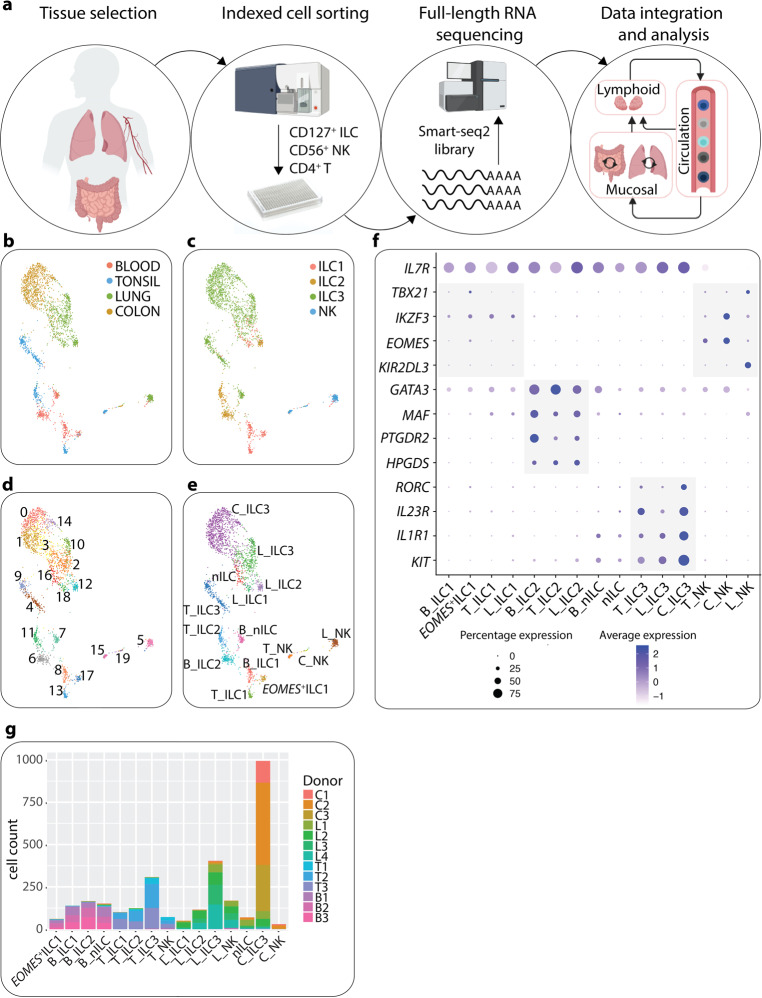
ScRNA-seq reveals subset and tissue-specific transcriptional imprinting. **a** Summary of the experimental workflow. Arrows in data integration and analysis section visualize the concept of immune cell migration between the tissues analyzed. Tonsil data was obtained from^12^ using the same scRNA-seq platform. **b** UMAP visualization of all cells (*n* = 2956) color-coded on the basis of tissue origin. **c** UMAP visualization of all cells, color-coded on the basis of cell surface phenotype inferred from indexed FACS sorting. **d** UMAP showing unbiased graph-based clustering analysis of all cells. **e** UMAP of annotated clusters. C_, colon; L_, lung; B_, blood; T_, tonsil; nILC, naïve ILC. **f** Dotplot displaying expression of selected and previously described human ILC subset-specific TFs and cell surface markers used to annotate clusters in **e**. **g** Barplot showing the representation of each donor in each annotated cluster in **e**. Data are from ten independent experiments with one tissue donor each (blood = 3, lung = 4 and colon = 3) integrated with data from.^12^

Transcriptional profiling of ILCs across tissues on the bulk^2,3^ or single-cell level^6,7,9^ in humans and mice have revealed unique features of ILCs that are dependent on the tissue microenvironment. Here we performed flow cytometric sorting of human CD127^+^ ILCs from three tissues representing two distinct immune cell compartments: circulation (blood), and mucosal tissue (lung and colon) (3–4 donors/tissue) (Fig. 1a; Supplementary Information Fig. S1). As reference populations we included selected NK populations along the spectrum of previously described tissue NK cell phenotypes. Predominantly recirculating CD56^dim^CD16^+^ NK cells were sorted from the lung^13^ and predominantly tissue-resident NK cells were sorted as bulk CD56^+^ from the gut^14^ or as CD56^bright^NKG2A^+^ NK cells from the tonsil.^12^ Cells were next processed for full-length Smart-seq2 scRNA-seq to unravel in-depth tissue-specific transcriptional profiles and heterogeneity. Our own previously published Smart-seq2 scRNA-seq dataset of CD127^+^ ILC and NK cells from tonsils,^12^ representing a SLO, was included in the analysis. All datasets were combined and analyzed together in order to identify common and distinct patterns across tissues and cell types (Fig. 1a). Low quality cells and a minor fraction of contaminating cells lacking expression of *PTPRC* (encoding CD45) and expressing transcripts of antigen-presenting cells (e.g., high expression of *HLA-DQA1*, *HLA-DRA* and HLA-*DPA1* at levels exceeding ILC) were identified and removed (Supplementary Information, Fig. S2), resulting in the analysis of a total of 2956 cells distributed across four tissues (Fig. 1b; Supplementary Information, Fig. S2).

Cell embedding using uniform manifold approximation and projection (UMAP)^15^ revealed that all ILC subsets as well as NK cells separated according to tissue of origin and FACS cell subset annotation as inferred from index sorting (Fig. 1b, c). Unbiased graph-based clustering subdivided cells into 20 clusters that segregated cells both by tissue of origin and FACS phenotype (Fig. 1d). On average, we observed 90% agreement between protein FACS phenotype and the clusters identified in scRNA-seq (Supplementary Information, Fig. S3a). We next used previously described human ILC subset-specific TFs and cell surface markers^12^ to annotate the clusters (Fig. 1e, f). Clusters 5 (lung), 15 (tonsil) and 19 (colon), expressing *NKG7*, *EOMES* and *GZMA/B* but lacking *IL7R* were annotated as NK cells. Clusters 8 (blood), 13 (tonsil), 18 (lung) expressed *IKZF3* (encoding the TF and zinc finger protein Aiolos) but lacked *EOMES*, and *KIR2DL1/3*, and were conditionally annotated as ILC1. Cluster 17 consisted of both tonsil and blood CD127^+^ ILCs, which expressed low levels of *EOMES*, conditionally annotating them as *EOMES*^+^ ILC1. Clusters 6 (blood), 11 (tonsil) and 12 (lung) were annotated as ILC2 on the basis of uniquely high *GATA3*, *MAF*, *PTGDR2* and *HPGDS* expression (Fig. 1e, f). As predicted by the low frequency of human colon ILC1 and ILC2 in non-inflamed colon tissue,^16^ colon cells with a FACS phenotype of ILC1 and ILC2 (total 10 cells) did not generate distinct clusters. ILC3s were annotated on the basis of *RORC*, *IL23R*, *IL1R1* and *KIT* expression (cluster 4 and 9 in tonsil, 2 and 10 in lung and 0, 1, 3 and 14 in colon) (Fig. 1e, f). We also observed *IL7R* and *KIT*-expressing ILCs in the blood (cluster 7) as well as in colon and lung (cluster 16), which lacked transcripts of all master TFs (Fig. 1e, f), reminiscent of the previously described ILC progenitors in blood and tissues.^12,17^ These cells were annotated as naïve ILCs (nILCs), consistent with the nomenclature for naïve T cells.^18^ Importantly, cells from all donors of each tissue were present in all annotated cell types (Fig. 1g) and clusters (Supplementary Information, Fig. S3b, c) for each tissue.

### Migration- and tissue-resident transcriptional profiles of ILCs

**Fig. 2:**
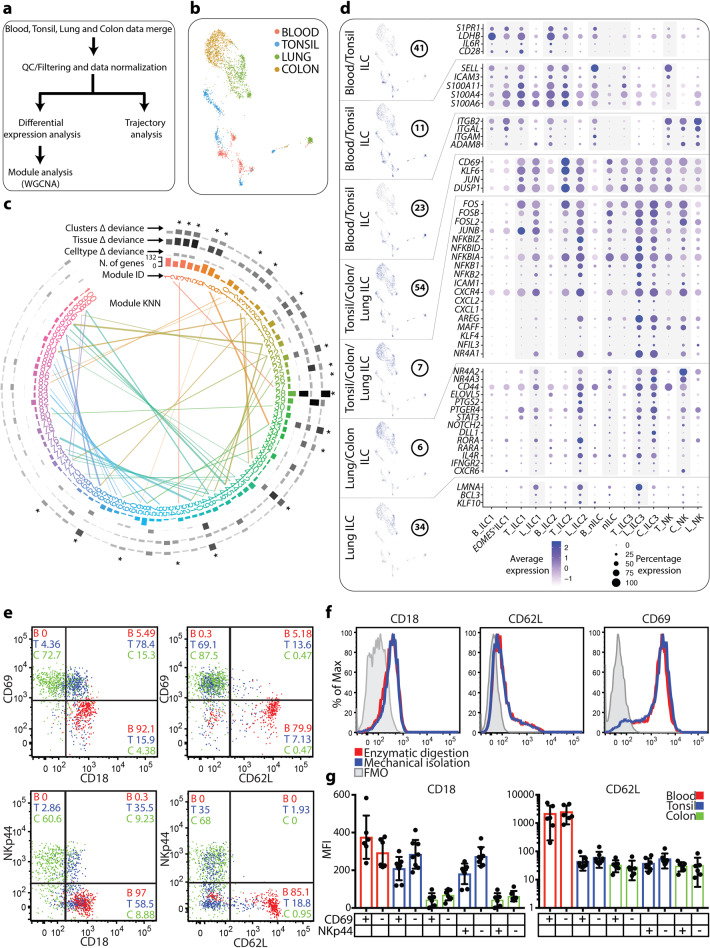
Migration and tissue-resident transcriptional profile of ILCs. **a** Summary of analysis approach used to identify gene modules and perform trajectory analysis. **b** UMAP visualization of all cells, color-coded on the basis of tissue origin. **c** Circular dendrogram illustrating the number and composition of each module and the contribution of each module to discriminating cell clusters, tissues and cell types (as inferred from FACS). Modules with highly correlated expressions are indicated with a 5-NN graph (connecting lines represent KNN; k nearest neighbor). Modules described in text are marked with an asterisk. **d** Modules highly correlated with tissues and/or cell types/clusters containing genes involved in migration or tissue residency. The figure shows a selection of genes per module, and their levels of expression within annotated clusters (Fig. 1e). Data in **a**–**d** is from ten independent experiments with one tissue donor each (blood = 3, lung = 4 and colon = 3) integrated with data from.^12^ **e** Co-expression of CD69 and CD18/CD62L (top row) and NKp44 and CD18/CD62L (bottom row) on total ILCs from blood, tonsil and colon biopsies. **f** Histogram showing the expression of CD18, CD62L and CD69 on mechanically or enzymatically isolated total tonsil ILCs. FMO, fluorescence minus one. **g** Bar plot quantification of the results shown in **e**. MFI, mean fluorescence intensity. Data in **e**–**g** are from six independent experiments with at least one tissue donor each (blood = 6, tonsil = 8 and colon = 6).

To identify conserved gene expression patterns that are shared across tissues and cell types, we used a two-step approach rather than simple differential expression (DE), which reveals differences but not similarities between cell types and tissues. DE gene lists for each annotated cell type and the overlaps between cell types and tissues are provided in Supplementary Information, Data S1. For the two-step approach, we selected the top DE genes in any of the 20 clusters (4410 genes) and subsequently performed weighted gene co-expression network analysis (WGCNA) for identification of 100 gene modules (see Materials and Methods for details) (Fig. 2a, b; Supplementary Information, Data S1). A high number of modules were chosen in order to avoid forcing genes with different expression patterns into the same group. Modules with highly correlated expression are indicated with a connecting line in the graph (Fig. 2c). Next, modules were scored individually for their contribution to discriminating tissues, cell types and/or cell clusters (Fig. 2c; Supplementary Information, Fig. S3d). Notably, gene modules did not contribute equally to explaining metadata and thus only modules that contributed to at least one cell type or tissue were selected for further in-depth analysis herein (Fig. 2c).

The analysis of four different tissues that represent unique immune niches (circulation, lymphoid- and mucosal tissue) in combination with the unbiased gene module analysis allowed us to identify conserved patterns across tissues and cell types (Fig. 2d). We identified two gene modules (41 and 11) containing transcripts highly correlated to the expression of the prototypical T cell migratory molecules *S1PR1* and *SELL* (encoding L-selectin; CD62L), respectively (Fig. 2d). Genes in these two modules were highly expressed in tonsil and blood ILCs, including nILCs. The modules included the glycolysis-associated transcript *LDHB*, the adhesion molecule transcript *ICAM3*, as well as transcripts for several S100 proteins (*S100A4*, *S100A6* and *S100A11*) (Fig. 2d), shown to be involved in migration.^19^ Module 23 was also over-represented in blood and tonsil and contained transcripts for several integrins including *ITGB2* (CD18) *ITGAL* (CD11a) and *ITGAM* (CD11b) (Fig. 2d). CD18 complexes with CD11 molecules to form the adhesion molecules lymphocyte function-associated antigen-1 (LFA-1), which together with CD62L are involved in endothelial tethering and firm adhesion. In sharp contrast to the migratory phenotype of blood and tonsil ILCs, we also identified gene modules indicative of tissue residency of lung and colon ILCs, including tissue nILCs and to some extent also of tonsil ILCs. Module 54 revealed a set of genes highly correlated to the expression of the prototypical tissue-residency-associated marker *CD69* (Fig. 2d). We confirmed the diametrically opposed migratory vs tissue-residency profiles of blood as compared to colon ILCs by demonstrating the absence of CD69 and high expression of CD18 and CD62L on blood ILCs and low expression of CD18 and CD62L and high expression of CD69 on colon ILCs (Fig. 2e). Tonsil showed a mixed phenotype, regardless of mechanic or enzymatic isolation of the cells (Fig. 2f), with low expression of CD62L, intermediate expression of CD18 and some cells expressing CD69, aligning with the presence of both tissue resident and migratory ILCs in secondary lymphoid tissues in mice.^20^ CD69^+^ tonsil ILCs expressed lower levels of CD18 as compared to CD69^–^ tonsil ILCs (Fig. 2g). Similarly, expression of NKp44 was associated with reduced expression of CD18 (Fig. 2g). These data not only validate the transcriptional findings but also show that in contrast to colon, tonsil CD69^+^ express the endothelial adhesion integrin CD18, as described for intravascular liver-resident T cells.^21^

Showing a similar expression pattern, we identified module 7 as containing genes previously reported in human tissue resident T cells^22^ involved in Fos/Jun signaling (*FOS*, *FOSB*, *FOSL2* and *JUNB*), NFκB signaling (*NFKBIZ*, *NFKBID*, *NFKBIA*, *NFKB1* and *NFKB2*), adhesion (*ICAM1*) and *CXCR4* (Fig. 2d). In this module were also transcripts of effector molecules such as *CXCL2*, *CXCL1* and *AREG*, the latter previously reported as an ILC2 effector molecule.^23^ The expression of *MAFF*, *KLF4*, *NFIL3* and *NR4A1* in tissue ILCs suggests that these TFs are involved in transcriptional programs of activation and/or tissue-residency in colon, lung and tonsil ILCs. Module 6, which was particularly expressed in mature but not nILCs in colon and lung, contained transcripts of additional nuclear receptor subfamily four group members, *NR4A2* and *NR4A3*, as well as the hyaluronic acid receptor *CD44*, previously shown to be expressed by tissue-resident T and NK cells^13,22^ (Fig. 2d). This module also contained transcripts involved in lipid metabolism (*ELOVL5* and *PTGS2*). The expression of *STAT3*, *NOTCH2*, *DLL1*, *RORA* and *RARA* suggests a potential role for STAT3-, Notch-, retinoic acid- and cholesterol-derivative (e.g., oxysterol) signaling in controlling activation and/or tissue-residency of colon and lung ILCs (Fig. 2d). Colon and lung ILC3, but to a lesser extent tonsil ILC3 expressed transcripts encoding the receptors for IL-4 (*IL4R*), IFN-γ (*IFNGR2*) and CXCL16 (*CXCR6*), raising the possibility that these factors are involved in the tissue-specific regulation of colon and lung ILC3. Furthermore, we identified module 34, containing lung-restricted transcripts indicative of tissue-residency^22^ including *LMNA* and the TFs *KLF10* and *BCL3* (Fig. 2d), the latter involved in NFκB signaling.

In summary, we identified three gene modules allowing for the characterization of the lymphoid- and peripheral tissue homing properties of tonsil and blood ILCs, including blood nILCs. Four additional modules contained transcripts for known and potential activation and/or tissue-residency associated proteins that showed tissue-restricted expression across colon, lung and tonsils. Most of these genes were highly expressed by colon and lung ILCs, including tissue nILCs, but to some extent also by tonsil ILCs. Importantly, these genes were not expressed by blood ILCs, including blood nILCs. Furthermore, lung NK^dim^ cells, which have been reported as circulating^13^ also showed low expression of transcripts known to be associated with tissue-residency.

### Gene modules reveal subset- and tissue-specific profiles of ILC3

**Fig. 3:**
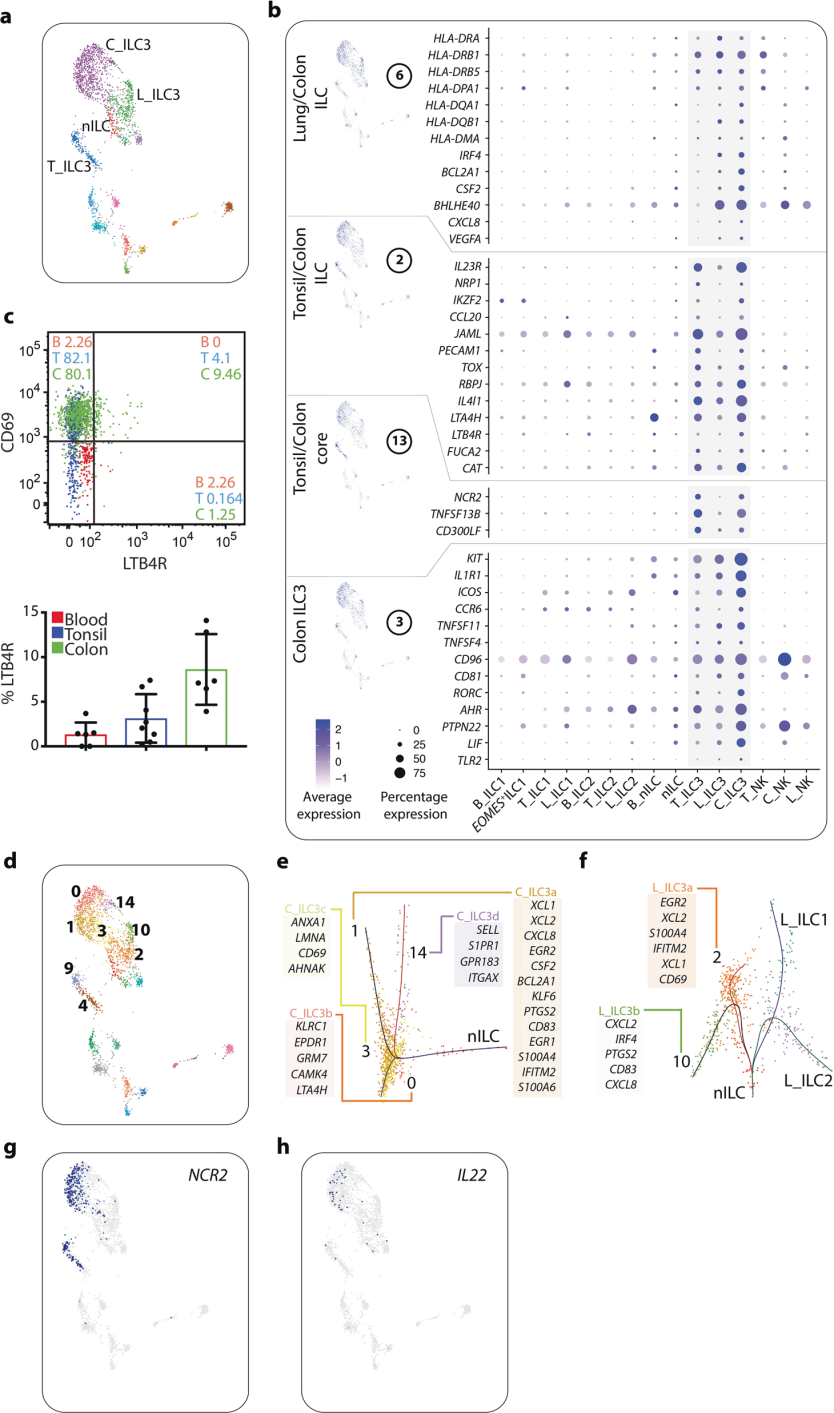
Tissue- and subset-specific profile of ILC3. **a** UMAP visualization of annotated ILC3 and nILC clusters (C_, colon; L_, lung; B_, blood; T_, tonsil; nILC, naïve ILC). Clusters are color coded based on annotated clusters (Fig. 1e). **b** Modules highly correlated with tissues and ILC3. The figure shows a selection of genes per module, and their levels of expression within annotated clusters (Fig. 1e). **c** Expression of LTB4R on colon ILC3 (top plot) and summary bar plot showing expression of LTB4R on colon ILC3, tonsil ILC3 and blood nILCs (bottom plot). Data in **c** (top) is from one representative blood, tonsil and gut sample. Data are from six independent experiments with one tissue donor each (blood = 6, tonsil = 8 and colon = 6). **d** UMAP visualization of unbiased graph-based clustering analysis of ILC3. Clusters are color coded based on unbiased graph-based clustering analysis of all cells (Fig. 1d). **e** Trajectory analysis of nILCs and colon ILC3 displaying a selection of DE genes for each colon ILC3 cluster (C_ILC3a–d). **f** Trajectory analysis of nILCs and lung ILC1, ILC2 and ILC3 displaying a selection of DE genes for each lung ILC3 cluster (L_ILC3a–b). **g** UMAP visualization of *NCR2* expression. Colored cells (shades of blue) indicate expression of *NCR2* transcripts. **h** UMAP visualization of *IL22* expression. Colored cells (shades of blue) indicate expression of *IL22* transcripts. Data in **a**, **b**, **d**–**h** is from ten independent experiments with one tissue donor each (blood = 3, lung = 4 and colon = 3) integrated with data from.^12^

Module 6 specified shared characteristics of putative tissue-resident ILCs in lung and colon (Fig. 2d). Notably, some of the genes in this module were particularly confined to ILC3 in these two tissues (Fig. 3a, b). These included transcripts encoding HLA-DR, -DP and -DQ (Fig. 3b), which we previously described in the tonsil.^12^ Additional transcripts in this module included *IRF4* and *BCL2A1*, indicating a potential role for these transcripts in establishing tissue-unique features of human colon and lung ILC3. Also the transcript for the effector cytokine GM-CSF, *CSF2* and *BHLHE40*, the latter a metabolism-related gene reported to regulate GM-CSF production in mouse ILC3,^24^ as well as *CXCL8* and *VEGFA* were expressed in lung and colon ILC3 (Fig. 3b), further indicating colon and lung-associated transcriptional imprinting of ILC3 as compared to tonsil ILC3. However, colon and tonsil ILC3 also shared striking similarities with respect to high expression of the prototypical ILC3 transcripts *NCR2*, *IL23R*, *NRP1*, *IKZF2*, *TNFSF13B*, *CCL20*, *JAML*, *PECAM1*, *TOX*, *RBPJ* and *CD300LF* as well as the previously described Th17 cell transcript *IL4I1* (Fig. 3b). Of interest, colon ILC3 expressed both *LTA4H*, encoding the leukotriene B4 (LTB_4_) forming enzyme LTA4-hydrolase, and the transcript coding for the receptor for LTB_4_, *LTB4R*, indicating a role for LTB_4_ in colon-specific regulation of ILC3 function. Further strengthening this hypothesis, a subset of colonic ILC3 expressed the LTB4R surface protein (Fig. 3c). A comparison of DE gene expression between lung, colon and tonsil ILC3 is provided as part of Supplementary Information, Data S1.

Module 3 contained ILC3-related genes that were expressed by ILC3 in all tissues but at particularly high level and frequency in colon ILC3 (Fig. 3b). These included surface molecule transcripts *KIT*, *IL1R1*, *ICOS*, *CCR6*, *TNFSF11* (encoding RANK ligand), *TNFSF4* (encoding OX40 ligand), the ITIM-containing *CD96* and the tetraspanin *CD81*. We also identified transcripts for the TFs *RORC* and *AHR*, the signaling molecule *PTPN22* and the cytokine *LIF* as preferentially highly expressed by colon ILC3 (Fig. 3b).

Finally, we evaluated the transcriptional profile of the clusters of cells that were termed nILCs in blood and tissues (colon and lung) on the basis of lack of markers associated with mature ILCs (Fig. 1f). From the evaluation of ILC3 (Fig. 3a, b) it became obvious that these cells show few transcriptional parallels to ILC3 despite their phenotypical similarities as assessed by flow cytometry. Expression of genes associated with migration and tissue-residency revealed a clear migratory behavior of blood nILCs whereas tissue nILCs showed features of tissue residency (Fig. 2d). A list of DE genes unique for blood and tissue nILCs is provided as part of Supplementary Information, Data S1. This list includes TFs of potential importance for the differentiation of nILCs in blood (e.g., *TCF7*) and lung/colon (e.g., *XBP1* and *BATF*) (Supplementary Information, Data S1).

In summary, we unveil the transcriptional features of nILCs in blood and tissues and reveal tissue-specific differences among ILC3 and potential novel functions of these cells by description of transcripts previously not known to be expressed by human ILC3.

### A spectrum of mucosal ILC3 transcriptional states branching in trajectory analysis

Next we performed trajectory analysis via Slingshot^25^ to understand the potential relationships between the putative nILCs identified in blood and tissues, and the more mature ILC subsets and the potential spectrum of activation states of ILC3 in colon and lung. Overlaying these trajectories with the clusters identified in Fig. 1d (Fig. 3d) not only illustrated the heterogeneity among ILC3 in colon, lung and tonsil but also inferred the potential transcriptional relationships between these transcriptionally distinct ILC3 subclusters (Fig. 3e, f; Supplementary Information, Fig. S4a, b). ILC3s were particularly heterogeneous in the colon where four subclusters could be identified through Louvain clustering (colon ILC3a–d, lung ILC3a–b, tonsil ILC3a–b) (Figs. 1d, 3e, f; Supplementary Information, Fig. S4a). Some of these clusters were dominated by, but not unique to, individual donors as cells from all donors of each tissue were present in all clusters (Supplementary Information, Fig. S3b, c) for each tissue. Of importance, the TF *IKZF2* and the master ILC3 TFs *RORC* and *AHR* were expressed across all ILC3 subclusters in all tissues, assigning them to the ILC3 lineage (Supplementary Information, Fig. S4c). Analysis comparing clusters within each tissue and focusing on the top 20 DE genes (Supplementary Information, Data S2), revealed that colon ILC3a (cluster 1) contained cells with an activated and potentially tissue-resident phenotype, expressing transcripts for multiple effector molecules involved in chemotaxis and recruitment of immune cells including *CSF2*, *XCL1*, *XCL2*, *CXCL8*, *S100A4* and *S100A6* (Fig. 3e). *EGR1*, *EGR2 and KLF6*, encoding TFs involved in immune cell activation,^26,27^ and *BCL2A1*, coding for an anti-apoptotic protein, were also enriched in this cluster of cells. The prototypical ILC3 cytokine *IL22* and the activation marker *NCR2* were expressed in colon ILC3a, as well as in colon ILC3b (cluster 0) (Fig. 3g, h); the latter also showed an activated phenotype with expression of genes involved in glutamate response and lipid metabolism, respectively (*GRM7* and *LTA4H*). Colon ILC3a was most similar to lung ILC3a (cluster 2) in terms of *XCL1*, *XCL2, S100A4, EGR2 and IFITM2*, but also shared similarities with lung ILC3b (cluster 10) in terms of *CXCL8, CD83* and *PTGS2* (encoding COX2) (Fig. 3e, f). ILC3s in the lung also branched into two subclusters, indicating distinct transcriptional trajectories which both contained features of activation and tissue residency (Fig. 3f). The activated phenotype of colon ILC3a stood in sharp contrast to colon ILC3d (cluster 14), which was most similar to lung ILC3b (Supplementary Information, Data S2). Colon ILC3d additionally expressed *SELL* (encoding CD62L), also expressed by tonsil ILC3b (cluster 4) (Supplementary Information, Fig. S4a),^12^ *S1PR1*, *ITGAX* and *GPR183* (Fig. 3e), indicative of a migratory behavior reminiscent of LTi-like cells contributing to colonic lymphoid structure formation in a GPR183-dependent manner in mice.^28^ Importantly, colon ILC3d formed a separate branch in trajectory analysis and were transcriptionally distinct from the tissue nILCs (Fig. 3e).

In summary, ILC3s show a high degree of transcriptional heterogeneity, in particular in the colon where we identified a spectrum of ILC3 states with variable activation status which separated into different branches in trajectory analysis, ranging from cells with a migratory phenotype, to ILC3-expressing effector molecules important for metabolic adaptations, tissue repair and immune cell recruitment.

### Gene modules reveal subset- and tissue-specific profiles of ILC2

**Fig. 4:**
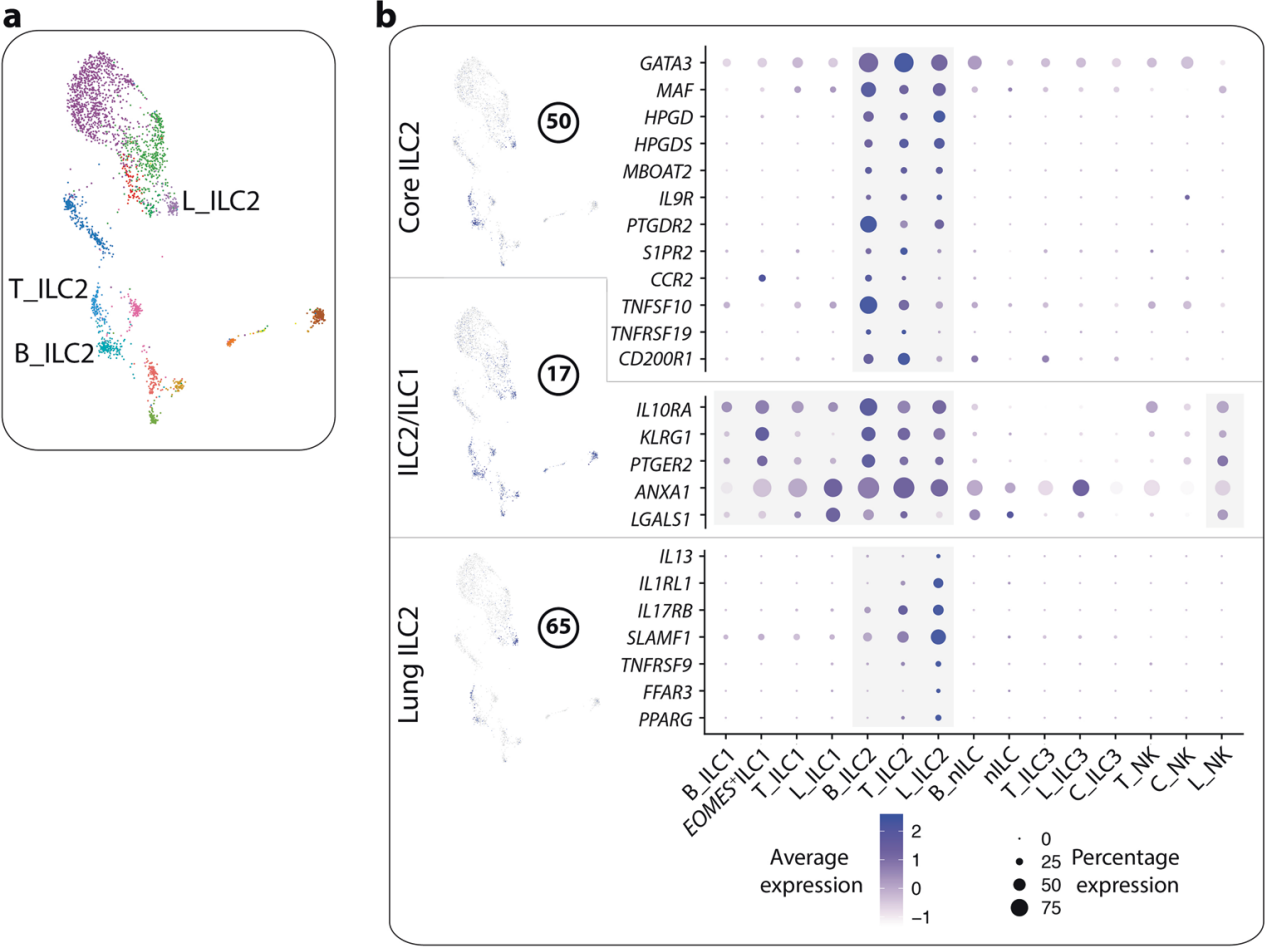
Tissue- and subset-specific profile of ILC2. **a** UMAP visualization of annotated ILC2 clusters (L_, lung; B_, blood; T_, tonsil). Clusters are color coded based on annotated clusters (Fig. 1e). **b** Modules highly correlated with ILC2 or tissue. The figure shows a selection of genes per module, and their level of expression within annotated clusters (Fig. 1e). Data are from ten independent experiments with one tissue donor each (blood = 3, lung = 4 and colon = 3) integrated with data from.^12^

Our gene module approach identified a set of genes that were specifically expressed in ILC2 across the three tissues (Fig. 4a, b), making up an ILC2 core transcriptome. These genes included, as expected, *GATA3* and *MAF*, as well as *HPGD*, *HPGDS* and *MBOAT2*, the latter three encoding proteins involved in lipid metabolism. Furthermore, ILC2 in all tissues expressed transcripts for the IL-9 receptor (*IL9R*). Other genes in the module were particularly expressed in blood ILC2 or blood and tonsil ILC2, including transcripts for receptors involved in cell trafficking and migration such as the prototypical ILC2 marker CRTH2 (*PTGDR2*), *S1PR2* and *CCR2*, the latter shown to be involved in the regulation of S1P receptors.^29^ The TNF family-related transcripts *TNFSF10* (encoding TRAIL) and *TNFRSF19* (encoding TROY) were also highly expressed by blood and tonsil ILC2, as well as *CD200R1*, possibly acting as an inhibitory receptor for human blood and tonsil ILC2. Another ILC2-related module was less restricted to ILC2 but still contained transcripts relevant for ILC2 biology, particularly for the potential negative regulation of these cells; *IL10RA*,^30^ *KLRG1*,^31^ *PTGER2*,^32^ *ANXA1*^33^ as well as *LGALS1*, encodes the immunosuppressive molecule galectin-1.

Finally, we identified a module of genes (number 65) specifically expressed in lung, but not tonsil or blood ILC2 (Fig. 4b). Expression of the main effector molecule *IL13* indicated that lung ILC2 are highly activated. Indeed, lung ILC2 showed high expression of *IL1RL1* and *IL17RB*, encoding the receptors for the ILC2-activating alarmins IL-33 and IL-25, respectively. Additional transcripts for potential activating receptors included the SLAM-family receptor *SLAMF1, TNFRSF9*, the fatty acid receptors *FFAR3* and *PPARG*, the latter shown to activate ILC2 in mice.^34^

**Fig. 5:**
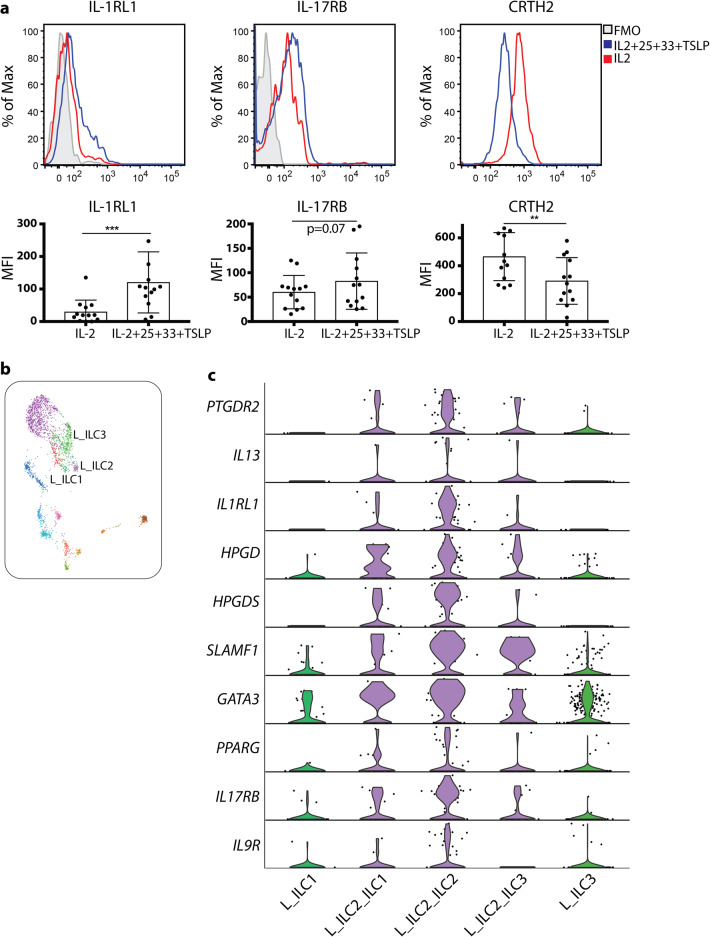
Exposure of blood ILC2 to alarmins recapitulates the lung ILC2 phenotype. **a** Expression of IL-1RL1, IL-17RB and CRTH2 protein on the surface of blood ILC2 treated with an alarmin cocktail (IL-2, IL-25, IL-33 and TSLP) for 48 h. FMO, fluorescence minus one. **b** UMAP visualization of annotated ILC clusters in the lung. Clusters are color coded based on annotated clusters (Fig. 1e). **c** Violin plot visualization of differentially expressed genes in cells annotated as ILC2 with a FACS phenotype of ILC1 (L_ILC2_ILC1) or ILC3 (L_ILC2_ILC3) vs cells ILC1 or ILC3, respectively. Data are from seven independent experiments with a total of 13 blood donors (**a**) or from ten independent experiments with one tissue donor each (blood = 3, lung = 4 and colon = 3) integrated with data from^12^ (**b**, **c**). Bars in **b** represent means ± SD. Mann Whitney test was used in **a**; **P < 0.01; ***P < 0.001.

The fact that blood ILC2 showed a phenotype associated with re-circulation and migration on both the transcriptional (Figs. 2d, 4b) and protein (Supplementary Information, Fig. S4d, e) level support the possibility that blood ILC2 might be recruited to the lung, as previously suggested in the mouse^35,36^ and in a human lung allergen-provocation study.^37^ We therefore hypothesized that exposure of blood ILC2 to an inflammatory type 2-deviating environment could induce the lung ILC2 phenotype. To address this possibility, we sorted and exposed blood ILC2 to the prototypical ILC2-activating cocktail of alarmins (IL-2, IL-25, IL-33 and TSLP) and assessed phenotypical changes (Supplementary Information, Fig. S5). Indeed, alarmin exposure increased the expression of IL-1RL1 and IL-17RB surface proteins on blood-derived ILC2 (Fig. 5a), phenocopying lung ILC2. In parallel, the expression of CRTH2 was downregulated, which we previously showed can be promoted by alarmin-mediated endogenous production of the CRTH2 ligand PGD_2_.^38^ This finding raised the possibility that in the lung, alarmin-activated ILC2 might not express CRTH2. Indeed, utilizing our indexed flow cytometry data, evaluation of the composition of the cluster annotated as lung ILC2 (Fig. 1d; Supplementary Information, Fig. S3a) revealed a relatively high presence (35%) of CRTH2^−^ ILCs (ILC1 and ILC3 by FACS phenotype) among the lung ILC2 which was not seen for blood or tonsil ILC2 where the majority of cells expressed CRTH2 protein (Fig. 1d; Supplementary Information, Fig. S3a). Evaluation of the transcriptional profile of CRTH2^–^ vs CRTH2^+^ cells showed that ILC2-related genes were present in CRTH2^– ^ILC2, and at higher levels and frequencies as compared to lung ILC1 and ILC3 (Fig. 5b, c).

In summary, we reveal tissue-specific transcriptional differences between ILC2 in blood, tonsil and lung particularly related to migration, tissue-residency and sensing of the tissue microenvironment. Exposure of blood ILC2 to an inflammatory type-2 environment lead to development of a lung-like phenotype including downregulation of CRTH2. Thus, our transcriptional data highlight heterogeneity among lung ILC2, specifically identifying CRTH2^−^ ILC2 with transcriptional similarities to bona fide CRTH2^+^ ILC2, including expression of the ILC2 effector molecule *IL13*.

### Gene modules reveal subset- and tissue-specific profiles of ILC1-like cells

**Fig. 6:**
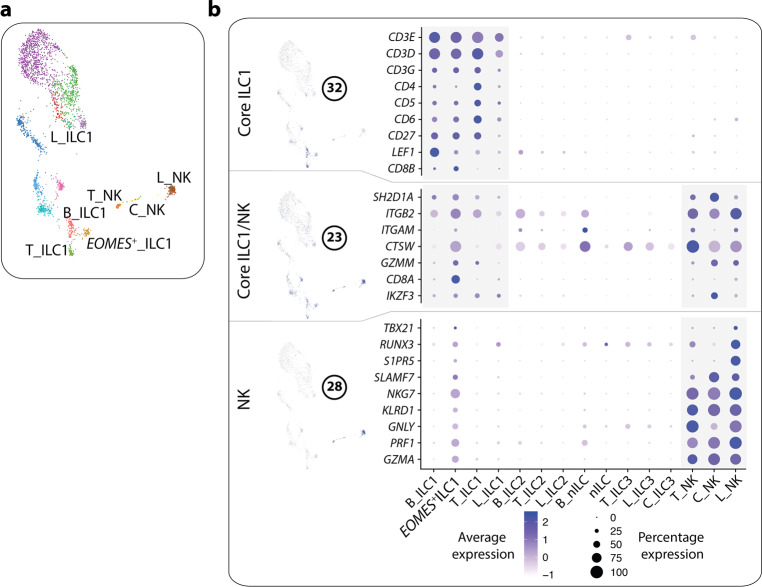
Tissue and subset specific profile of ILC1. **a** UMAP visualization of annotated ILC1 and NK cell clusters. Clusters are color coded based on annotated clusters (Fig. 1e). **b** Modules highly correlated with ILC1 and NK cells. The figure shows a selection of genes per module, and their level of expression within annotated clusters (Fig. 1e).

Module 32 contained transcripts which were uniquely expressed by putative ILC1 and *EOMES*^+^ ILC1 in blood and tonsil (Fig. 6a, b). In line with previous reports,^12,39^ the module consisted of T cell-related genes including *CD3D, CD3E, CD3G, CD4, CD5*, *CD6 and CD27*. *LEF1*, a TF transcript described in tonsil ILC1,^12^ was also expressed by blood ILC1. Notably, the transcripts in this module were expressed by few lung ILC1 suggesting that blood and tonsil ILC1 have more pronounced T cell features than lung ILC1 (Fig. 6b). Furthermore, indicating that blood and tonsil ILC1, and in particular *EOMES*^+^ ILC1, are more related to NK cells than lung ILC1, two additional transcriptional modules (Fig. 6b) revealed transcripts expressed in NK cells as well as in ILC1 and *EOMES*^+^ ILC1 in blood and tonsil, and to a lesser extent by ILC1 in lung (Fig. 6b).

### Widespread predicted TCR V(D)J-rearrangement in blood ILC1-like cells

**Fig. 7:**
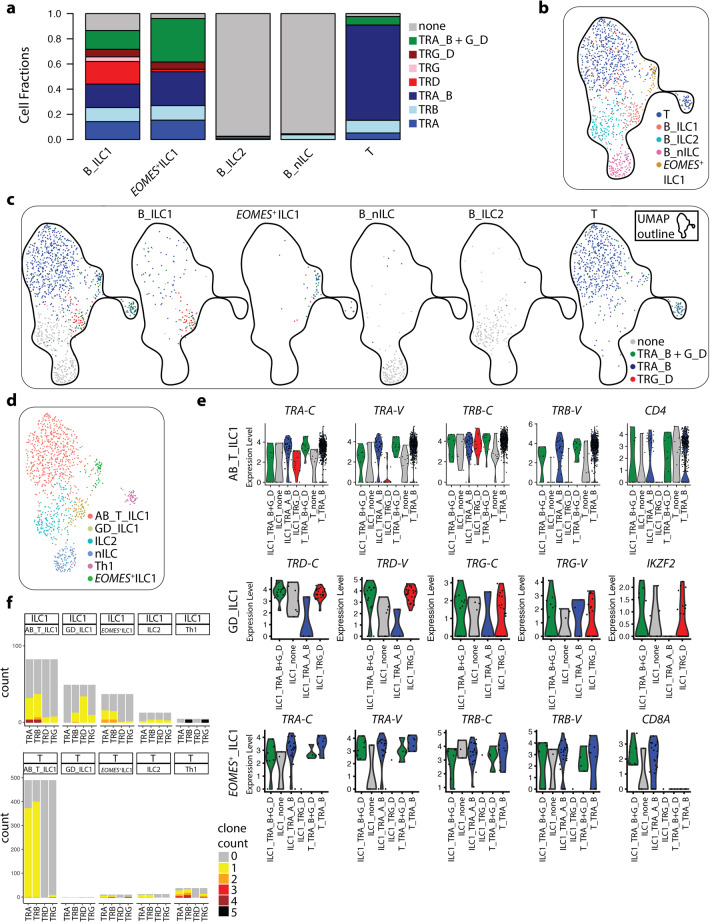
Rearrangement of V(D)J T cell receptor genes in blood ILCs and T cells. **a** Predicted V(D)J-rearrangement of *TRA*, *TRB*, *TRD* and/or *TRG* genes in annotated blood ILC clusters (B_ILC1, EOMES^+ ^ILC1, B_ILC2 and B_nILC) and in donor matched CD4^+^ T cells (T). TRA_B, rearrangement of *TRA* and *TRB* genes; TRG_D, rearrangement of *TRG* and *TRD* genes; TRA_B + G_D, rearrangement of *TRA* and/or *TCRB* in combination with *TRD* and/or *TRG* genes. **b** UMAP of blood ILCs and CD4^+^ T cells color-coded on the basis of annotation in Fig. 1e (B_ILC1, *EOMES*^+^ ILC1, B_ILC2 and B_nILC) and CD4^+^ T cells (T). **c** UMAP displaying each subset in **b** separately and color-coding the cells on the basis of predicted V(D)J-rearrangement of *TRA* and/or *TRB* (TRA_B), *TRG* and/or *TRD* (TRG_D) or *TRA* and/or *TCRB* in combination with *TRD* and/or *TRG* genes (TRA_B + G_D). **d** Annotation of clusters on the basis of cell composition and predicted TCR V(D)J-rearrangement pattern. **e** Violin plot representation of selected DE genes in AB_T_ILC1 cluster (top), GD_ILC1 (middle) and *EOMES*^+^ ILC1 (bottom). Within each cluster, data is displayed separately for ILC1 and T cells, and also on the basis of TCR V(D)J-rearrangement pattern. **f** Number of cells with different clone frequencies (based on third complementarity region 3 (CDR3)) per rearranged TCR chain for all ILC1 (top) and CD4^+^ T cells (bottom) in the different clusters in **d**. Data are from three independent scRNA-seq experiments with one blood donor each. Total number of cells is 1036 (536 CD4^+^ T cells and 473 ILCs).

Since putative ILC1 and *EOMES*^+^ ILC1 in blood and tonsil showed characteristics of T cells, we performed a direct comparison between blood ILCs and CD4^+^ T cells sorted in parallel from the same peripheral blood donors (Supplementary Information, Figs. S1 and S6). To understand the T cell identity of ILC1, we analyzed TCR V(D)J-rearrangement using MiXCR.^40^ In contrast to the T cell population, which as expected predominantly consisted of cells with rearranged TCR-α and -β chains, ILC2 and nILCs showed minimal V(D)J rearrangement across any of the TCR chains (Fig. 7a; Supplementary Information, Fig. S7a). This stood in sharp contrast to putative blood ILC1 and *EOMES*^+^ ILC1, where most of the cells had undergone TCR rearrangement (Fig. 7a; Supplementary Information, Fig. S7a), despite lacking surface expression of TCR α/β, γ/δ or CD3 (Supplementary Information, Fig. S1a). While *EOMES*^+^ ILC1 mostly had predicted rearrangement in α- and/or β-TCR chains, blood ILC1 showed predicted rearrangement in all four TCR chains (Fig. 7a; Supplementary Information, Fig. S7a). UMAP clustering revealed that blood ILC1 with α- and/or β-TCR rearrangement chains clustered with CD4^+^ T cells (AB_T_ILC1) (Fig. 7b–d; Supplementary Information, Fig. S7b, c). DE analysis showed that while the majority of ILC1 expressed transcripts of α- and/or β-TCR constant regions (*TRA-C* and *TRB-C*), expression of *CD4* as well as variable fragments of α- and/or β-TCR genes (*TRA-V* and *TRB-V*) was more pronounced in ILC1 with α- and/or β-TCR rearrangement (Fig. 7e; Supplementary Information, Fig. S7d, e). ILC1 with rearrangement in predominantly δ-, but also γ-TCR chains formed a distinct cluster (GD_ILC1) (Fig. 7c, d; Supplementary Information, Fig. S7b) and concordantly expressed fragments of γ- and/or δ-TCR variable chains (*TRG-V* and *TRD-V*) as well as *IKZF2* (Fig. 7e; Supplementary Information, Fig. S7d, e). In contrast, *EOMES*^+^ ILC1, of which most had predicted rearrangements in α/β chains, clustered with T cells in close proximity to CXCR3^+^ Th1 cells^41^ (Fig. 7b–d; Supplementary Information, Fig. S7c). DE analysis unveiled that *EOMES*^+^ ILC1 with predicted rearrangement in α/β chains showed high expression of α- and/or β-TCR constant and variable regions, as well as *CD8A* (Fig. 7e; Supplementary Information, Fig. S7d, e). Hence, *EOMES*^+^ ILC1 are transcriptionally closely related to Th1 and cytotoxic T cells. Finally, we evaluated the clonality of ILCs vs T cells. As expected from healthy donor blood, most cells represented unique clones (Fig. 7f; Supplementary Information, Fig. S7f). Among ILC1 and *EOMES*^+^ ILC1 with α/β rearrangement, there were six clones represented by two or more cells (Fig. 7f; Supplementary Information, Fig. S7f). Two of these clones were shared with T cells (Supplementary Information, Fig. S7g).

In summary, in contrast to ILC2 and nILCs, the majority of blood ILC1 and *EOMES*^+^ ILC1 are strikingly similar to CD4^+^ or CD8^+^ TCR α/β^+^ T cells, respectively, in terms of expression profile, TCR rearrangement and clonality. In contrast, blood ILC1 with preferential δ-TCR rearrangement constitute a subset distinct from T cells. Our conclusions were further strengthened when comparing the transcriptional profiles of ILCs to blood CD3^+^ T cells and CD56^+^ NK cells in a publically available scRNA-seq data set (Supplementary Information, Fig. S8), showing minimal similarity between blood ILC1 with preferential δ-TCR rearrangement and any of the T cell subsets (Supplementary Information, Fig. S8e, f).

## Discussion

Previous reports have described tissue-specific transcriptional imprinting of ILCs across multiple organs. However, these findings originated from pre-defined ILC subsets sorted in bulk from mice^2^ or humans,^3^ neglecting heterogeneity in the sorted populations. Here we used full-length scRNA-seq on individually sorted ILCs allowing for integrated analysis of FACS phenotype and transcriptome on the single-cell level. Additionally, we took a holistic and unbiased approach to analyzing the scRNA-seq data by complementing DE analysis with correlation analysis to identify gene modules, thereby going beyond only describing markers specific to each cell type. Through our approach, we have been able to make a number of novel observations, accompanied by a set of experimental functional studies, which advance our understanding of human ILC biology and serve as a resource to the immunology community.

We identified two distinct ILC clusters that lacked markers of mature ILCs. In the blood, these cells expressed CD117, identifying them as the previously described circulating progenitors of all lineages of mature ILCs, including NK cells.^4,17^ The other cluster consisted of predominantly CD117^+^ ILC from colon and lung. In sharp contrast to circulating nILCs, these cells showed a transcriptome compatible with tissue-residency, possibly representing a resident precursor to mature ILCs in these tissues. nILCs are identified as ILC3 by flow cytometry,^3^ calling for a better cell surface definition of these cells. Future studies should be aimed at targeted sorting and enrichment of these cells based on e.g., expression of CD117 and lack of markers associated with mature ILC subsets and activation such as CRTH2, HLA-DR and NKp44. This might reveal nILC subsets polarized to specific fates in certain tissues. Our in-depth unbiased transcriptional profiling of circulating and resident nILCs provide a road-map for such differentiation studies of these cells and their role in disease conditions.

Parabiosis experiments in mice have suggested that ILCs in mucosal and adipose tissues are largely tissue resident^42^ while other reports have unveiled a recirculating behavior of colon ILC2 during *N. brasiliensis* infection.^36^ We identified gene modules particularly enriched in naïve and mature ILCs in the colon and lung including *CD69* and transcripts encoding TFs (*NR4A1-3*, *STAT3*, *RORA* and *RARA*) as well as transcripts involved in Notch-, Fos/Jun- and NFκB signaling, many of which have previously been detected in CD69^+^ T cells and NK cells in humans and mice.^13,22^ In sharp contrast to colon and lung, naïve and mature blood ILCs showed features of CD62L- and S1PR1-mediated recirculation, similar to that described in mice.^36^ In the tonsil, we detected signs of both recirculation and tissue-residency. These data align well with a recent report on human ILC3 sorted in bulk from tonsils, spleen and blood.^4^ Additionally, in mice, lymph nodes harbor both migratory and tissue-resident ILCs, the former more common for ILC1.^20^ Although the tissue-residency and migratory profiles of mouse ILCs seem largely conserved in humans, we did detect some unique features of human ILCs in this regard. Most strikingly, human ILC2 uniquely expressed *S1PR2*, opening up for the possibility of selectively targeting this receptor to influence ILC2 migration during type 2 inflammation.

The colonic mucosa is continuously exposed to environmental and intrinsic factors derived from the microbiota, diet and secretions in the digestive system. Indeed, we detected the highest expression of ILC3-related genes in this tissue, and also the highest degree of heterogeneity among ILC3. These ILC3 represented a spectrum of cells ranging from those with a more resting and recirculating phenotype, possibly constituting cells recently recruited from the circulation, to cells expressing genes encoding effector molecules, activation markers and molecules involved in metabolism. These data align well with a recent scRNA-seq study of ILCs in the mouse colon,^8^ although the precise transcripts defining ILC3 subsets in the mouse colon was different to those we identified here. Notably, we did not identify ILC1-biased ILC3, as those recently reported in the human inflamed colon,^11^ which is likely due to the non-inflamed nature of the intestinal tissue in our analysis. Furthermore, our data confirm previous reports that in stark contrast to the mouse, the human healthy colon lacks ILC2 as defined by surface protein expression.^16,43^ Our data now further conclusively demonstrate that the human colon is also largely devoid of ILCs with a transcriptome reminiscent of ILC2. These results provide a blueprint of the homeostatic situation of ILCs in the human colon, aiding studies of the role for ILCs in colon immunopathology.

In the lung, we identified ILC2 with a transcriptome indicative of activation, including expression of receptors of both IL-25 and IL-33, reminiscent of natural ILC2 described in mice.^44^ The lung-specific transcriptome of ILC2 could be a result of the constant exposure of the lung to both extrinsic and intrinsic immune stimuli. Indeed, we could recapitulate the lung phenotype of ILC2, with high expression of receptors for IL-33 and IL-25, and low expression of CRTH2, by exposing circulating ILC2 to tissue-derived alarmins. This indicates that activated ILC2 might not necessarily express CRTH2, which has previously been suggested based on both mouse^35^ and human ex vivo^37,45^ and in vitro data.^38^ Utilizing the power of our flow cytometry indexed scRNA-seq data we were indeed able to identify such CRTH2^–^ ILC2 in the lung, expressing receptors for IL-33 and IL-25. In addition to downregulation of CRTH2 on ILC2, CRTH2^–^ ILC2 might also be derived from ILC1 and/or ILC3 transdifferentiation. However, such ILC2 plasticity from bona fide ILC1 or ILC3 has, to the best of our knowledge, never been demonstrated in either mice or humans. Nevertheless, our unbiased identification of CRTH2^–^ ILC2 challenges our understanding of human ILC2 biology, and has consequences for the identification of activated human ILC2 and strategies to target these cells in type 2 inflammation in e.g., allergy and asthma. The failure of the CRTH2 antagonist Fevipiprant in moderate-to-severe asthma^46,47^ may, at least in part, be explained by our findings. The expression of the cell surface receptor SLAMF1 could be helpful to distinguish CRTH2^–^ ILC2 from ILC1 and ILC3 in the lung.

The existence of ILC1 in different human tissues in homeostasis and inflammation has been a topic of much debate.^48–51^ While Tbet^+^ non-NK/T/B lymphocytes, fulfilling the definition of ILC1, clearly accumulate in inflamed human intestinal tissues,^16,52,53^ Simoni et al. have suggested that in non-affected human tissues, ILC1s are artifacts derived from contamination, by e.g., T cells. In partial support of this, Roan et al. showed that a fraction (7%) of blood ILC1 upregulate surface expression of TCRα/β following a 7-day culture.^39^ While this shows that indeed blood ILC1 might contain a small fraction of T cells that have downregulated their surface TCRs, the majority of blood ILC1 are likely not T cells in disguise despite expressing intracellular CD3 epsilon.^39^ In fact, CD3 epsilon overexpression in the absence of TCR expression in mice has been suggested to block T cell development.^54^ Furthermore, cytoplasmic CD3 epsilon expression might indicate that blood ILC1 are immature, as it is observed in fetal but not adult NK cells.^55^ In further support of that, CD5^+^ ILCs, expressing intracellular CD3 epsilon, have been suggested as precursors of mature IFN-γ-producing ILC1.^56^ We identified a particularly pronounced T-cell signature, including *CD3E*, encoding CD3 epsilon, in putative ILC1 and *EOMES*^+^ ILC1 in blood and tonsil. We therefore analyzed TCR rearrangement to better understand ILC1-T cell relationships. *EOMES*^+^ ILC1 and a subset of ILC1 showed pronounced rearrangement in TCRα/β chains as well as expression of *CD4* and *CD8A*, respectively. Despite lacking surface expression of TCRs and CD3, these ILCs might very well be T cell contaminants, which was also supported by partial clonal overlap between TCRα/β-rearranged T cells and ILC1. Clonal overlap was however uncommon, and therefore it is also possible that these ILCs are “failed T cells” with non-productive TCRα/β- rearrangement, leading to a lack of surface TCR expression. Notably, in ILCs, the expression of TCRα/β variable chains was associated with TCRα/β rearrangement and could potentially be used as a proxy for rearrangement in these cells.

We also identified a cluster of ILC1, which was transcriptionally distinct from T cells, with rearrangement in predominantly TCR-δ chains. Indeed, mouse NK cell precursors show V(D)J-recombination activity^57^ and up to a third of human blood NK cells display rearrangement of δ-TCR chains.^58^ Hence, similar to intracellular CD3 epsilon expression,^55^ δ-chain rearrangement in blood ILCs might be a sign of immaturity and a history of recombination enzyme activity.

In summary, our comparisons of ILCs and CD4^+^ T cells in human blood unveiled clusters of putative ILC1 that show TCRα/β-rearrangement and clonal overlap with T cells. The latter are likely T cell contaminants with down-regulated surface TCRs while the remainder could be “failed T cells”, with non-productive TCR rearrangement. We also identified ILCs with TCR-δ rearrangement reminiscent of immature ILCs, rather than differentiated Tbet^+^ ILC1. Our findings provide important first steps towards more extensive, elaborate studies on rearrangement in subsets of T and ILCs/NK cells across the innate and adaptive landscape and the mechanisms, including development, underlying this unexpected TCR rearrangement.

In conclusion, we used scRNA-seq to identify a plethora of ILC subsets ranging from circulating and tissue-resident nILCs to subsets of mature, activated and tissue-resident ILCs with altered metabolism across four tissues. Deepened analysis unveiled the presence of lung-specific CRTH2^−^ ILC2, challenging our understanding of ILC2-mediated diseases. Furthermore, TCR-V(D)J rearrangement studies identified widespread predicted TCRα/β-rearrangement and a distinct subset of putative immature ILCs among the ILCs commonly referred to as ILC1 in peripheral blood. Our findings constitute a roadmap for future studies aimed at understanding tissue-specific ILC-mediated immunity in immunopathology.

## Materials and methods

### Human tissue samples

The Swedish ethical review authority approved all studies of human tissues and all studies were performed according to the Declaration of Helsinki. All regulations for work with human tissues were complied with. All study subjects gave their informed written consent prior to tissue collection.

### Cell isolation from lung tissue

Lung tissue was obtained from four patients undergoing lobectomy for lung cancer (age 57–76 year old, three females, one male) at Karolinska University Hospital, Sweden. Non-affected tissue was obtained distally from the tumor. None of the patients had a diagnosis of asthma or chronic obstructive pulmonary disease (COPD). All patients were ex-smokers. None of the patients had received preoperative chemo- or radiotherapy. Lung cells were isolated from human lung tissue by a sequential combination of washing, enzymatic digestion, mechanical disruption and density centrifugation using Percoll gradient medium (WEMP protocol), as recently published by us^59^ where all details of the protocol can be found. Briefly, freshly resected lung tissue was washed in PBS and cut into smaller pieces, then enzymatically digested while stirring. The resulting digested product was cut and mechanically disrupted by syringe plunging and filtering. The filtered cell suspension was washed in PBS, resuspended in 30% Percoll (one phase) and centrifuged. The cell pellet was resuspended and red blood cells were lysed and washed with PBS 2% fetal calf serum (FCS) and cells were counted. The cell suspension was then stained with fluorescence-labeled antibodies for FACS.

### Cell isolation from whole blood

Peripheral blood mononuclear cells (PBMCs) were isolated from whole venous blood from three healthy donors (age 29–32 year old, two males, one female). 27 mL of fresh blood was collected in EDTA tubes and directly diluted 1:1 in PBS. PBMC were isolated via lymphoprep (Axis Shield) and gradient centrifugation for 20 min at 2000× rpm. Lymphocytes were then collected, washed and counted. The cell suspension was then stained with fluorescence-labeled antibodies for FACS.

### Cell isolation from colon biopsies

Biopsies from the ascending colon were obtained from three tumor-screening patients (age 57–71 year old, two males, one female) with known genetic predisposition for colon cancer (hereditary nonpolyposis colorectal cancer (HPNCC) or Lynch Syndrome) but without any polyps or tumors at the time of sampling. Colonoscopies were performed at Karolinska University Hospital or GHP Stockholm Gastro Center, Sweden. Biopsy retrieval and cell isolation was performed as previously described,^16^ where all the details of the protocol can be found. Briefly, eight punch-biopsies per patients were collected and transported in wash buffer, consisting of Hank’s Balanced Salt Solution (HBSS) supplemented with antibiotics, fungizone and HEPES. Samples were processed within 1 h of biopsy retrieval. Wash buffer was removed and biopsies were enzymatically digested while stirring. The filtered cell suspension was washed by centrifugation and the total cell pellet was used for staining with fluorescence-labeled antibodies for FACS.

### Tonsil data

The previously published tonsil raw data used in this study can be found at GEO: GSE70580; Sequence Read Archive: SRP060416.

### Cell sorting

Isolated cells were incubated with live/dead cell marker (DCM; Invitrogen) and surface protein antibodies (Supplementary Information, Table S1) at 4 °C for 30 min, washed with PBS and resuspended in Iscove’s modified Dulbecco’s medium IMDM medium supplemented with 1% normal human serum (NHS; Invitrogen) and Yssels supplement (made in house). Cells were sorted into 384-well plates (Eppendorf) containing 2.3 μL of lysis buffer per well. Lysis buffer (per well) consisted of 1.150 μL of 0.4% Triton X-100 (Sigma), 0.4 μL dNTPs mix (25 mM) (Thermo Life Tech), 0.05 μL Smart-dT_30_VN (100uM) (IDT), 0.05 μL RNAse inhibitor (40 U/μL) (Takara), 0.0025 μL ERCC (1:40,000 dilution) (Ambion) and 0.6475 μL of water (Thermo Life Tech). Cells were sorted on an Aria FUSION sorter (BD Biosciences), with FACS Diva version 8 software and indexed data analysis enabled. Sorting of blood ILC2 were performed on a SONY MA900 Multi-Application cell sorter.

### In vitro cultures

Sort-purified peripheral blood ILC2 (Supplementary Information, Fig. S5) were cultured in IMDM (Fisher Scientific) supplemented with 2% NHS and Yssels supplement in a 96 well plate. Cells were left in culture for 48 h at 37 °C with IL-2 (10 U/mL) or IL-2 (10 U/mL), IL-25 (R&D), IL-33 (PeproTech) plus TSLP (PeproTech) (at 50 ng/mL each). Following culture, the cells were washed and stained with fluorescence-labeled antibodies for flow cytometry.

### Flow cytometry

For flow cytometry analysis, cells were stained with DCM and antibodies (Supplementary Information, Table S1) for 30 min at 4 °C. Cells were washed in PBS supplemented with 2 mM EDTA and 2% FCS and fixed with 2% paraformaldehyde for 10 min at room temperature. Cells were acquired on an LSR Fortessa flow cytometer (BD Biosciences) with FACSDiva software version 8. Data were analyzed with FlowJo software version 9.9.6 (Tree Star).

### Preparation of cDNA libraries and sequencing

Sorted cells were processed using the Smart-seq2 protocol with minor changes.^60^ Sequencing was done to the average depth of 0.7 million reads per cell with 43 bp reads (single end) after demultiplexing. Blood samples were additionally sequenced for V(D)J analysis to and average depth of 0.84 million reads per cell with 150 bp reads (paired end) after demultiplexing.

### Read alignment and gene-expression estimation

The reads were aligned to the human genome (hg19–GRCh38) and spike-in sequences (from the External RNA Controls Consortium) using STAR v2.5.3a,^61^ and filtered for uniquely mapping reads. Counts per gene were calculated for each transcript in Ensembl release 92 using rpkmforgenes.^62^

### Data filtering and normalization

Bioinformatics analyses were performed using R programming language, using Seurat v3 package^63^ with modifications. Reads were normalized to transcripts per million reads (TPMs) in order to compensate for gene length in transcript count estimation. Values were then multiplied by the read length (43 bp) and rounded to the nearest integer. Cell libraries were considered of low quality and filtered out if: (1) the percentages of mitochondrial and ribosomal gene families were above 40% or 50%, respectively; (2) the percentage of protein-coding genes was below 50%; (3) the number of unique genes in a cell was below 200. Genes detected in less than five cells were excluded from the analysis. Counts were next log-normalized with scale factor of 1000 and Z-score transformed for regression of confounding factors such as effects related to mitochondrial, ribosomal, Y chromosome, cell cycle, sex and proportion of non-coding RNA.

### Dimensionality reduction and clustering

Normalized data were further processed for identification of highly variable genes using the Scran method^64^ and linear data compression using principal component analysis (PCA) from which the top 50 principal components (PCs) explaining about 70% of the variance in the dataset were chosen for downstream analysis. Non-linear dimensionality reduction was run on the top 50 PCs using UMAP^15^ and embedded into two final dimensions. Graph construction was also run on the top 50 PCs using approximate nearest neighbor (KNN)^65^ search with k = 10, reflecting the minimum number of cells expected to be considered a potentially rare cell cluster. Spurious connections were filtered using shared nearest-neighbors (SNN) search using Jacquard’s index of 1/15. Unsupervised graph clustering was run using the Louvain method, in which the modularity resolution parameter (res = 2.4) was optimized to match both known sample metadata information as well as a coherent cluster distribution across the UMAP embedding.

### Differential expression and gene module identification using WGCNA

Identification of marker genes associated to specific cell clusters was done using differential expression via MAST.^66^ For standard differential expression log_2_FC  > 0.25 and false discovery rate (FDR) < 0.01 was used.

For identification of the most relevant genes across cell clusters to be later used for weighted gene correlation network analysis (WGCNA),^67^ a permissive threshold with absolute log_2_FC > 0.1 and *P* value < 0.1 was used. Briefly, the 4410 genes identified in this manner were used for calculation of gene-to-gene Pearson correlations. The adjacency matrix was directly used for creation of the topological overlap matrix (TOM) using again Pearson as correlation measure. Power transformation of adjacency values were ignored since it would result in flattening all values to near 0, as even strong single-cell correlations rarely reach close to 1 due to the nature of data sparsity. No significant differences in the results were found when using Pearson and Spearman correlations, and therefore Pearson correlations were kept due to its faster overall performance. Gene module identification was done with agglomerative hierarchical clustering on the TOM using Ward’s squared linkage (Ward.D2) with a fixed number of 100 modules. Gene counts belonging to the same module were normalized between 0–1 individually and then averaged per cell for calculation of each gene module expression pattern.

Modules were prioritized by fitting a general linear model (GLM) using each gene module expression pattern as the effect and the sample metadata as explaining variable. Each module and metadata parameter was tested independently. The output of the GLM fit results in the residual metric of “deviance” ($dev_{model}$) after fitting the model and “null deviance” ($dev_{null}$) when considering a NULL model. The percent difference in these two deviances (${\Delta}dev$) was used as a measure of how much a particular metadata factor explains the expression pattern of a gene module:

$${\Delta}dev = \frac{{dev_{null} - dev_{model}}}{{dev_{null}}}$$

This way, modules with higher ${\Delta}dev$ were prioritized for further exploration throughout the manuscript. Modules enriched for ribosomal, mitochondrial and other known housekeeping families were mentioned but not further analyzed.

### Trajectory inference analysis

Trajectory inference analysis was performed using Slingshot v.1.2.0^25^ on the Diffusion Map embedding (using five components), since the UMAP embedding creates a distorted representation of the original space. Lineages were defined by setting the naïve clusters (nILC) as the root, and curves were projected onto the Diffusion Map embedding for visualization.

### TCR rearrangement analysis

For blood data with 150 bp PE sequencing, MiXCR^40^ was run on all cells using the “analyze shotgun” command and default settings. To avoid false positives MiXCR output was filtered for clones with count > 5. If multiple predicted clones were found, only dominant predicted clone from each chain (TRA, TRB, TRD or TRG) was included in downstream analysis.

### Analysis of human 10× genomics scRNA-seq PBMC data

Human PBMC data with 10× Genomics scRNA-seq, cell surface protein detection with antibody-derived tags (ADT) and V(D)J assay was downloaded from the 10× genomics website (https://support.10xgenomics.com/single-cell-vdj/datasets/3.0.0/vdj_v1_hs_pbmc2_5gex_protein). Dimensionality reduction and clustering was performed with Seurat v3.1.3 with the whole dataset. T-cell and NK-cell clusters were selected based on the protein expression, e.g. CD3^+^ and CD56^+^CD3^–^ clusters were selected for further analysis. Clustering and dimensionality reduction was performed again with this subset, as shown in Supplementary Fig. 8a, b and cell types annotated based on top DEGs using MAST (Supplementary Information, Fig. S8c). At this stage, a contaminating cluster 8 that is low in CD3 and CD56, but high in CD16 and CD19, was removed as it likely represented doublets or cells that were wrongly clustered in the first round.

To compare the 10× PBMC clusters to our blood SS2 data, we looked at intersections of DEGs. All significant upregulated DEGs (*P* < 0.01) for each cluster within the 10× data and SS2 data were calculated separately. In the case of the AB_T_ILC1 cluster, cells were separated into T-cells (AB_T) and ILCs (AB_ILC) and DEG analysis was performed separately for those two groups. For each comparison, phyper test *P* values were calculated for overlap between the gene lists with all genes expressed in five cells in both datasets as background.

For correlation between 10× PBMC and blood SS2 data, top 100 DEGs (or fewer if not 100 significant genes) from both datasets were used to calculate mean expression for each cluster. Spearman correlations between the clusters in each dataset were then calculated for the mean expression.

## Supplementary information

Supplementary Text

Supplementary Figure S1

Supplementary Figure S2

Supplementary Figure S3

Supplementary Figure S4

Supplementary Figure S5

Supplementary Figure S6

Supplementary Figure S7

Supplementary Figure S8

Supplementary Data S1

Supplementary Data S2

Supplementary Table S1

## Data Availability

Data produced in this manuscript is available on Gene Expression Omnibus (GEO) with accession number GSE150050.

## Code availability

All scripts used for the analysis can be found at https://github.com/czarnewski/ilc_tissue_imprinting.
