# Supplementary material for: Tissue-specific transcriptional imprinting and heterogeneity in human innate lymphoid cells revealed by full-length single-cell RNA-sequencing
Source: Cell Res. 2021 Jan 8;31(5):554–68. doi: 10.1038/s41422-020-00445-x (PMC8089104; doi:10.1038/s41422-020-00445-x)
Supplement: Supplementary file 1 — Supplementary Text [file 41422_2020_445_MOESM1_ESM.docx]

**Figure S1. Gating strategy for sorting of blood, colon and lung cells**

(**a**-**c**) Gating strategy for sorting of ILCs, NK cells or T cells from blood (**a**), lung (**b**) and colon (**c**).

LinDCM: lineage cocktail plus dead cell marker (DCM). Lineage cocktail contained antibodies against CD1a, CD14, CD19, CD34, CD94, CD123, BDCA2, FcεR1, TCRαβ, TCRγδ with (**b**) or without (**a**, **c**) CD3.

Data is from 10 independent experiments with one tissue donor each (blood=3, lung=4 and colon=3).

**Figure S2. Filtering of cells**

1. UMAP visualization of the data before filtering color-coded by unbiased graph-based clusters. Cluster 12, 16 and 20 were filtered out of the analysis.
2. UMAP visualization of *PTPRC* transcripts (encoding for CD45).
3. Dotplot displaying expression of the top 10 differentially expressed genes for cluster 12, 16 and 20 respectively. Four known ILC transcripts were also included in the plot.

Data is from 10 independent experiments with one tissue donor each (blood=3, lung=4 and colon=3).

**Figure S3. Confusion matrix and delta deviance scores**

1. Matrix showing the agreement between unbiased graph-based clustering (columns) and cell surface phenotype as inferred by FACS indexed data (rows). The discordance between cluster and FACS phenotype is shown as a percentage in the bottom row. The % discordance is calculated by dividing the number of cells deviating from the main FACS ILC celltype, by the number of total cells in the cluster, and multiplying the result by 100.
2. Matrix showing the number of cells per donor in each cluster.
3. Bar graph showing the number of cells per donor in each cluster.
4. Bar graph showing the contribution (delta deviance) of each module (1-100) to discriminating clusters, tissues, cell types, donors and plates respectively. Modules described in text are marked with an asterisk.

Data is from 10 independent experiments with one tissue donor each (blood=3, lung=4 and colon=3) integrated with data from ^12^.

**Figure S4. Slingshot trajectory analysis of tonsil and *ex vivo* isolated blood, tonsil and lung ILC2 CD69 and CD45RA protein expression.**

(**a**-**b**) Slingshot trajectory analysis of tonsil (**a**) and blood (**b**) ILCs displaying a selection of DE genes for each tonsil ILC3 cluster (T_ILC3a-b).

(**c**) Dotplot showing the expression of the top 20 DE genes per ILC3 subcluster in colon, lung and tonsil.

Data is from 10 independent experiments with one tissue donor each (blood=3, lung=4 and colon=3) integrated with data from ^12^.

(**d**-**e**) Expression of CD69 and CD45RA protein on *ex vivo* isolated blood, tonsil and lung ILC2. ILC2 were selected in a singlet-lymphocyte gate on the basis of FSC/SSC and subsequently gated as follows: DCM (dead cell marker)^-^CD45^+^lineage (CD1a, CD14, CD19, CD34, CD94, CD123, BDCA2, FcεR1, TCRαβ, TCRγδ)^-^CD3^-^CD127^+^CD161^+^CRTH2^+^.

Data is from 3-5 independent experiments with 3 tissue donors each (CD69, n=5 for all tissues; CD45RA, blood and lung=4, tonsil=3). Bars in (**d**) and (**e**) represent mean values ± SD. Mann Whitney test *p≤ 0.05, **p≤ 0.01.

**Figure S5. Gating strategy for sorting of blood ILC2**

ILC2 were FACS sorted in a singlet-lymphocyte gate on the basis of FSC/SSC and subsequently gated as follows: DCM (dead cell marker)^-^CD45^+^lineage (CD1a, CD14, CD19, CD34, CD94, CD123, BDCA2, FcεR1, TCRαβ, TCRγδ)^-^CD3^-^CD127^+^CD161^+^CRTH2^+^.

Data is from 7 independent experiments with a total of 13 blood donors

**Figure S6. Gating for T helper cell subsets (indexed data)**

CD4^+^ T cells were FACS sorted in a singlet-lymphocyte gate on the basis of FSC/SSC and defined as CD3^+^CD4^+^CD8^-^. Additionally, FACS indexed data of chemokine receptor expression was used to define T follicular helper cells (Tfh), Th1, Th17_Th1, Th2, Th9, Th17, Th22, naïve T cells (Tn) and three subsets of undefined T cells (U1-3).

Data is from three independent experiments with one blood donor each.

**Figure S7. TCR V(D)J rearrangement analysis of blood ILCs and T cells**

1. Predicted V(D)J-rearrangement of *TRA*, *TRB*, *TRD* and/or *TRG* genes in annotated blood ILC clusters (B_ILC1, EOMES^+^ILC1, B_ILC2 and B_nILC in **Figure 1e**) and in donor matched CD4^+^ T cell subsets gated according to Figure S6 (Tfh: T follicular helper cells, Tn: naïve T cells, U1-3: unidentified 1-3. TRA_B: rearrangement of *TRA* and *TRB* genes, TRG_D: rearrangement of *TRG* and *TRD* genes, TRA_B+G_D: rearrangement of *TRA* and/or *TCRB* in combination with *TRD* and/or *TRG* genes.
2. UMAP visualization of all blood ILCs and CD4^+^ T cells color-coded on the basis of unbiased graph-based clustering annotation.
3. UMAP visualization of blood ILCs and CD4^+^ T cells color-coded on the basis of T helper subset annotation as inferred from FACS indexed data in **Figure S6**.
4. Dotplot showing the expression of the top 10 DE genes between ILC1 (cells annotated as B_ILC1 merged with *EOMES*^+^ILC1 in **Figure 1e**) and T cells as inferred from FACS indexed data. Gene expression is displayed separately depending on TCR (V(D)J rearrangement pattern. TRA_B: rearrangement of *TRA* and/or *TRB* genes, TRG_D: rearrangement of *TRG* and/or *TRD* genes, TRA_B+G_D: rearrangement of *TRA* and/or *TCRB* in combination with *TRD* and/or *TRG* genes.
5. Dotplot showing the expression of the top DE genes for each cluster as annotated in **Figure 7d** (Th1, nILC, ILC2, AB_T_ILC1, GD_ILC1 and *EOMES*^+^ ILC1 (bottom).
6. UMAP visualization of blood ILCs (cells annotated as B_ILC1 merged with *EOMES*^+^ILC1 in **Figure 1e**) and CD4^+^ T cells (annotated from FACS indexed data, Figure S6). Cells are color-coded on the basis of CDR3-region clone count and shape-coded by cell type.
7. Sankey diagram showing the overlap of CDR3 clone identity for TRA, TRB, TRG and celltype (ILCs annotated by clustering in Figure 1e and T cells on FACS phenotype inferred from indexed data). Only cells with clone count >= 2 for any rearranged chain is included.

Data is from three independent scRNAseq experiments with one blood donor each.

**Figure S8. 10x data integration**

1. UMAP with clustering of 10x PBMC data (pre-selected CD3^+^ and CD56^+^CD3^-^ cells) with 8 clusters.
2. UMAPs with cell surface protein detection with antibody derived tags (ADT)
3. UMAP with cluster 8 removed and annotated by celltype.
4. Dotplot with top DEGs for each of the clusters.
5. Differentially expressed genes (DEGs) overlap for blood SS2 (x-axis) and 10x PBMC (y-axis) data. All DEGs between clusters within each of the datasets were run separately. The numbers in gray boxes at bottom/right part is the number of DEGs (only upregulated genes) per cluster and numbers in coloured boxes is the common genes to two lists. Colors of the boxes are -log10(p-value) from phyper test for significance of overlap. Cells in the AB_T_ILC1 cluster were separated into T-cells (AB_T) and ILCs (AB_ILC) for this analysis.
6. Heatmap with pairwise spearman correlation between clusters in the SS2 data (x-axis) and the 10x PBMC data (y-axis). Mean expression per cluster was calculated using the union of all top 100 upregulated DEGs per cluster from both datasets and used to calculate the correlations.

**Data S1. DE genes across all cells and tissues divided by modules. DE analysis with intra and inter tissue analysis across annotated clusters.**

See separate excel file: Data S1

**Data S2. DE genes in ILC3 subclusters in colon, lung and tonsil**

See separate excel file: Data S2

**Table S1. Antibodies used in the study**

See separate excel file: Table S1.
