# Supplementary material for: Tissue-specific transcriptional imprinting and heterogeneity in human innate lymphoid cells revealed by full-length single-cell RNA-sequencing
Source: Cell Res. 2021 Jan 8;31(5):554–68. doi: 10.1038/s41422-020-00445-x (PMC8089104; doi:10.1038/s41422-020-00445-x)
Supplement: Supplementary file 2 — Supplementary Figure S1 [file 41422_2020_445_MOESM2_ESM.pdf]

Figure S1

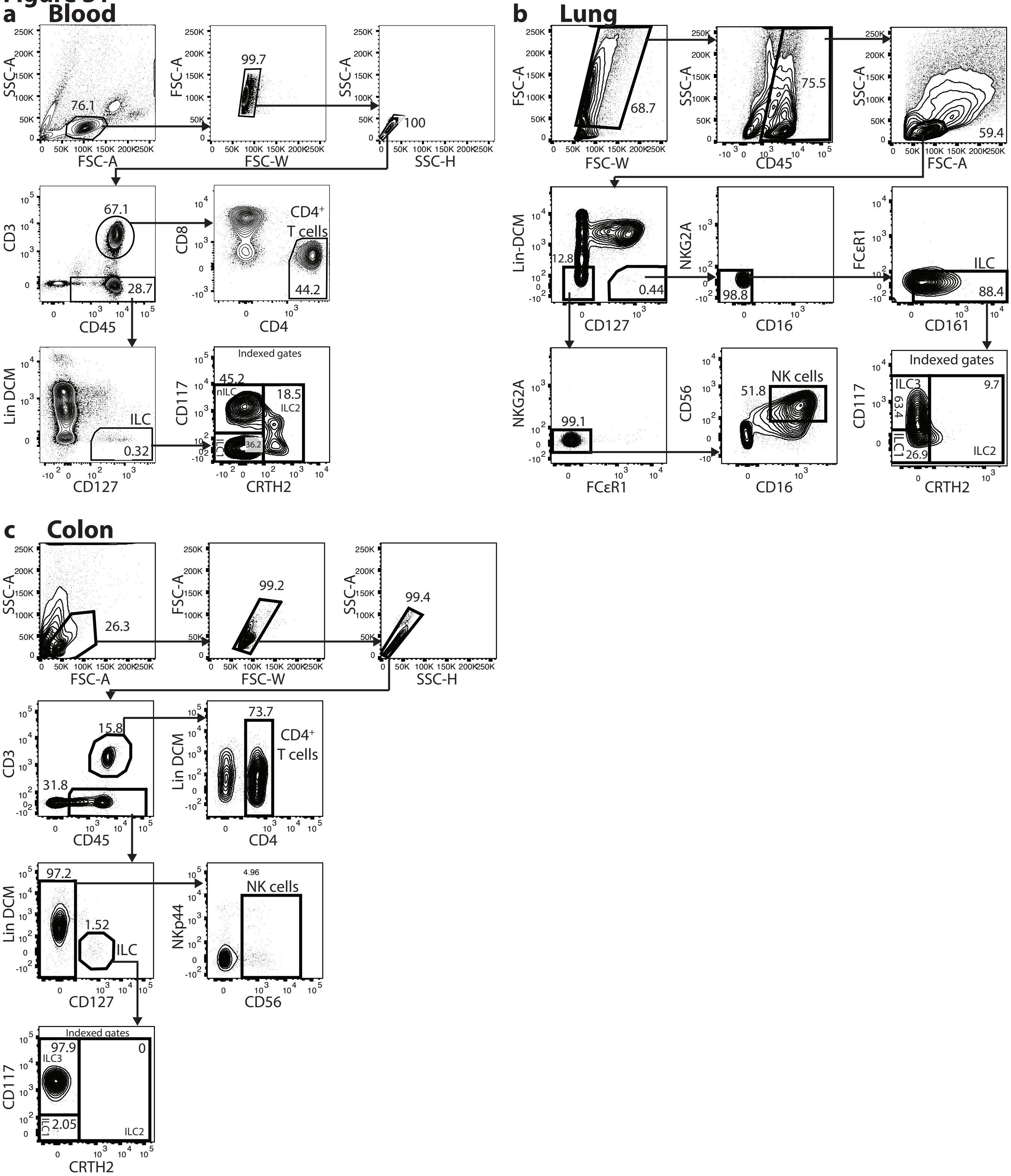

a Blood
b Lung
c Colon
SSC-A
FSC-A
FSC-W
SSC-H
CD3
CD45
CD8
CD4
CD4⁺ T cells
Lin DCM
Lin-DCM
CD127
ILC
CD117
CRTH2
Indexed gates
nILC
ILC1
ILC2
ILC3
NKG2A
CD16
FCεR1
CD161
CD56
NK cells
NKp44

Figure S1. Gating strategy for sorting of blood, colon and lung cells

(**a**-**c**) Gating strategy for sorting of ILCs, NK cells or T cells from blood (**a**), lung (**b**) and colon (**c**).

LinDCM: lineage cocktail plus dead cell marker (DCM). Lineage cocktail contained antibodies against CD1a, CD14, CD19, CD34, CD94, CD123, BDCA2, FcεR1, TCR$\alpha\beta$, TCR$\gamma\delta$ with (**b**) or without (**a**, **c**) CD3.

Data is from 10 independent experiments with one tissue donor each (blood=3, lung=4 and colon=3).
