# Supplementary material for: Tissue-specific transcriptional imprinting and heterogeneity in human innate lymphoid cells revealed by full-length single-cell RNA-sequencing
Source: Cell Res. 2021 Jan 8;31(5):554–68. doi: 10.1038/s41422-020-00445-x (PMC8089104; doi:10.1038/s41422-020-00445-x)
Supplement: Supplementary file 3 — Supplementary Figure S2 [file 41422_2020_445_MOESM3_ESM.pdf]

Figure S2

a

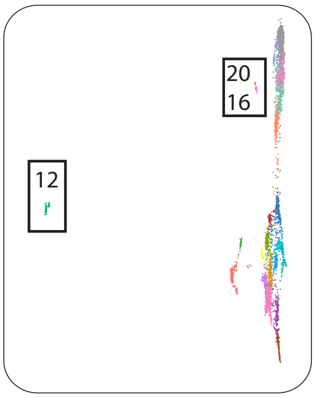

b

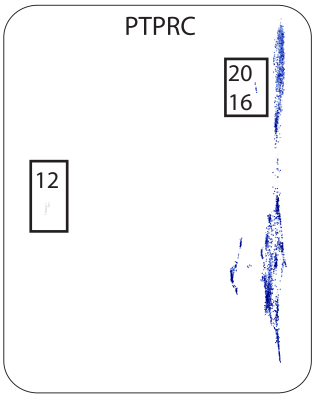

c

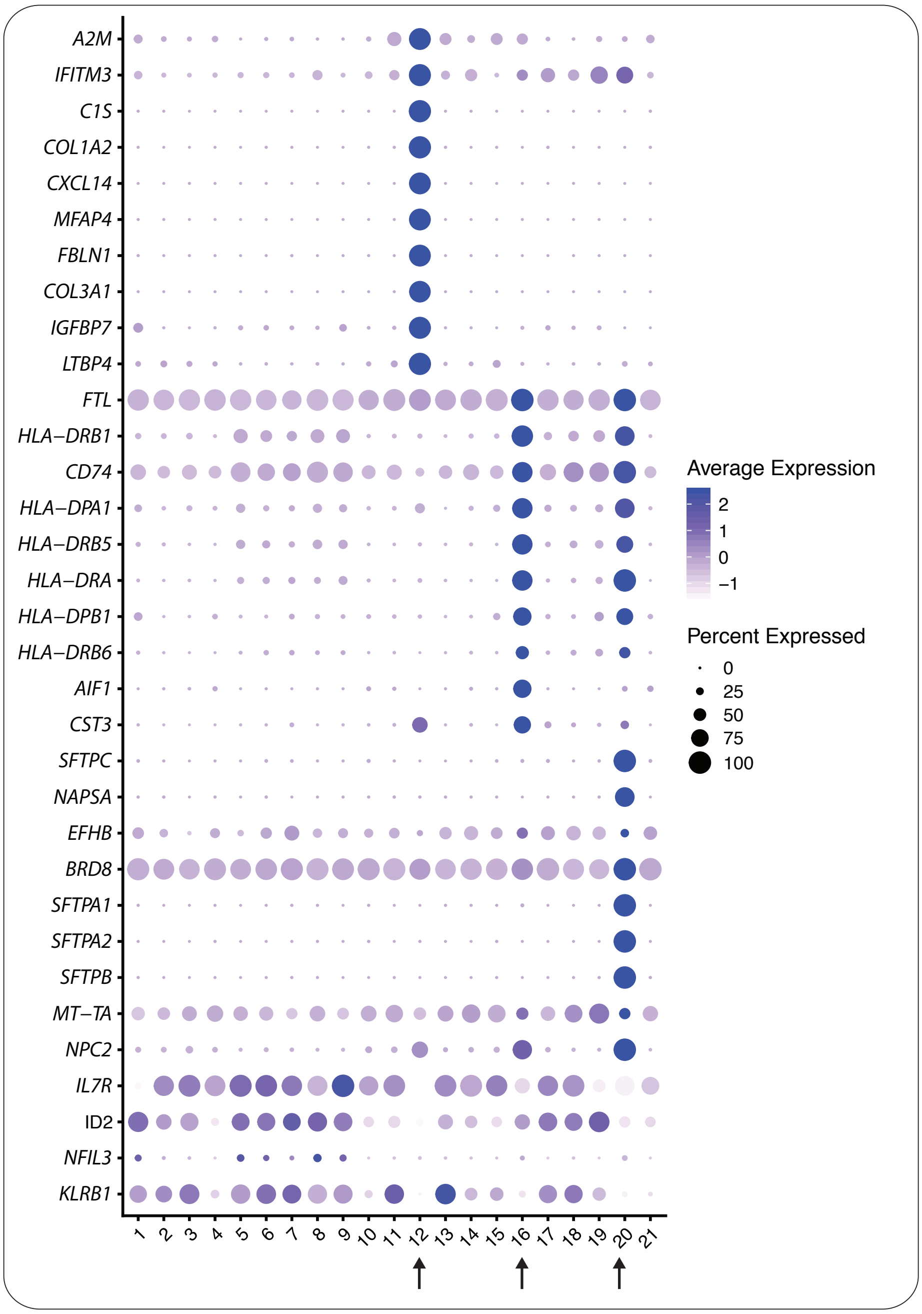

Figure S2. Filtering of cells

a) UMAP visualization of the data before filtering color-coded by unbiased graph-based clusters. Cluster 12, 16 and 20 were filtered out of the analysis.

b) UMAP visualization of *PTPRC* transcripts (encoding for CD45).

c) Dotplot displaying expression of the top 10 differentially expressed genes for cluster 12, 16 and 20 respectively. Four known ILC transcripts were also included in the plot.

Data is from 10 independent experiments with one tissue donor each (blood=3, lung=4 and colon=3).
