# Supplementary material for: Tissue-specific transcriptional imprinting and heterogeneity in human innate lymphoid cells revealed by full-length single-cell RNA-sequencing
Source: Cell Res. 2021 Jan 8;31(5):554–68. doi: 10.1038/s41422-020-00445-x (PMC8089104; doi:10.1038/s41422-020-00445-x)
Supplement: Supplementary file 4 — Supplementary Figure S3 [file 41422_2020_445_MOESM4_ESM.pdf]

Figure S3

a

	0	1	2	3	4	5	6	7	8	9	10	11	12	13	14	15	16	17	18	19
**BLOOD_ILC1**	0	0	0	0	1	8	4	7	128	0	0	0	0	0	0	5	2	47	0	1
**BLOOD_ILC2**	0	0	0	0	0	0	142	7	6	0	0	0	0	0	0	1	0	3	0	0
**BLOOD_ILC3**	0	0	0	0	4	0	9	118	0	0	0	0	0	0	0	2	0	2	0	0
**COLON_ILC1**	1	0	0	1	0	0	0	0	0	0	0	0	0	1	0	0	0	1	1	0
**COLON_ILC2**	0	0	0	0	0	0	2	0	0	0	0	0	2	0	1	0	0	0	0	0
**COLON_ILC3**	345	312	14	161	1	0	0	5	0	0	8	0	0	0	64	0	15	0	2	0
**COLON_NK**	0	0	0	0	0	2	0	0	0	0	0	0	0	0	2	0	0	0	1	28
**LUNG_ILC1**	0	0	31	3	1	0	0	2	1	0	1	1	18	1	0	0	9	1	36	1
**LUNG_ILC2**	0	1	2	2	0	0	1	0	0	0	2	5	74	0	0	0	6	0	1	0
**LUNG_ILC3**	11	19	223	60	2	0	1	7	0	0	121	1	22	0	7	0	38	0	9	0
**LUNG_NK**	0	0	0	0	1	159	0	0	0	0	0	0	0	0	0	0	0	0	0	0
**TONSIL_ILC1**	0	0	0	0	7	0	0	1	4	1	0	5	0	96	0	2	0	7	0	0
**TONSIL_ILC2**	0	0	0	0	5	0	7	1	0	1	0	110	0	3	0	0	0	0	0	0
**TONSIL_ILC3**	1	1	1	0	145	0	0	4	0	131	0	2	0	0	2	0	0	0	0	0
**TONSIL_NK**	0	0	1	0	7	0	0	0	0	1	0	0	0	0	0	62	0	0	0	0

**% discordance**	0,28	0,30	12,50	2,64	12,64	4,73	8,43	11,84	4,32	2,24	2,27	7,26	34,48	2,97	3,95	13,89	24,29	8,20	26,00	6,67

b

	0	1	2	3	4	5	6	7	8	9	10	11	12	13	14	15	16	17	18	19
**C1**	56	43	3	22	0	0	0	2	0	0	1	0	1	0	8	0	1	0	0	7
**C2**	270	49	6	112	1	2	1	2	0	0	4	0	1	1	54	0	12	0	4	20
**C3**	20	220	5	28	0	0	1	1	0	0	3	0	0	0	5	0	2	1	0	1
**L1**	1	6	44	38	1	32	1	3	0	0	2	1	5	1	1	0	34	1	6	0
**L2**	7	14	71	20	2	41	0	1	0	0	2	4	47	0	1	0	6	0	29	1
**L3**	0	0	86	6	0	39	1	2	0	0	31	0	25	0	2	0	8	0	6	0
**L4**	3	0	55	1	1	47	0	3	1	0	89	2	37	0	3	0	5	0	5	0
**T1**	0	0	0	0	25	0	0	3	0	11	0	9	0	0	1	20	0	0	0	0
**T2**	0	0	1	0	134	0	1	1	3	7	0	62	0	38	0	20	0	4	0	0
**T3**	1	1	1	0	5	0	6	2	1	116	0	46	0	61	1	24	0	3	0	0
**B1**	0	0	0	0	5	7	28	58	51	0	0	0	0	0	0	8	2	17	0	0
**B2**	0	0	0	0	0	0	56	37	42	0	0	0	0	0	0	0	0	25	0	0
**B3**	0	0	0	0	0	1	71	37	41	0	0	0	0	0	0	0	0	10	0	1

c

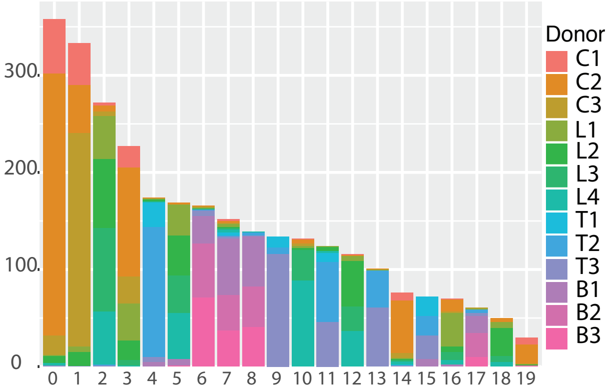

d

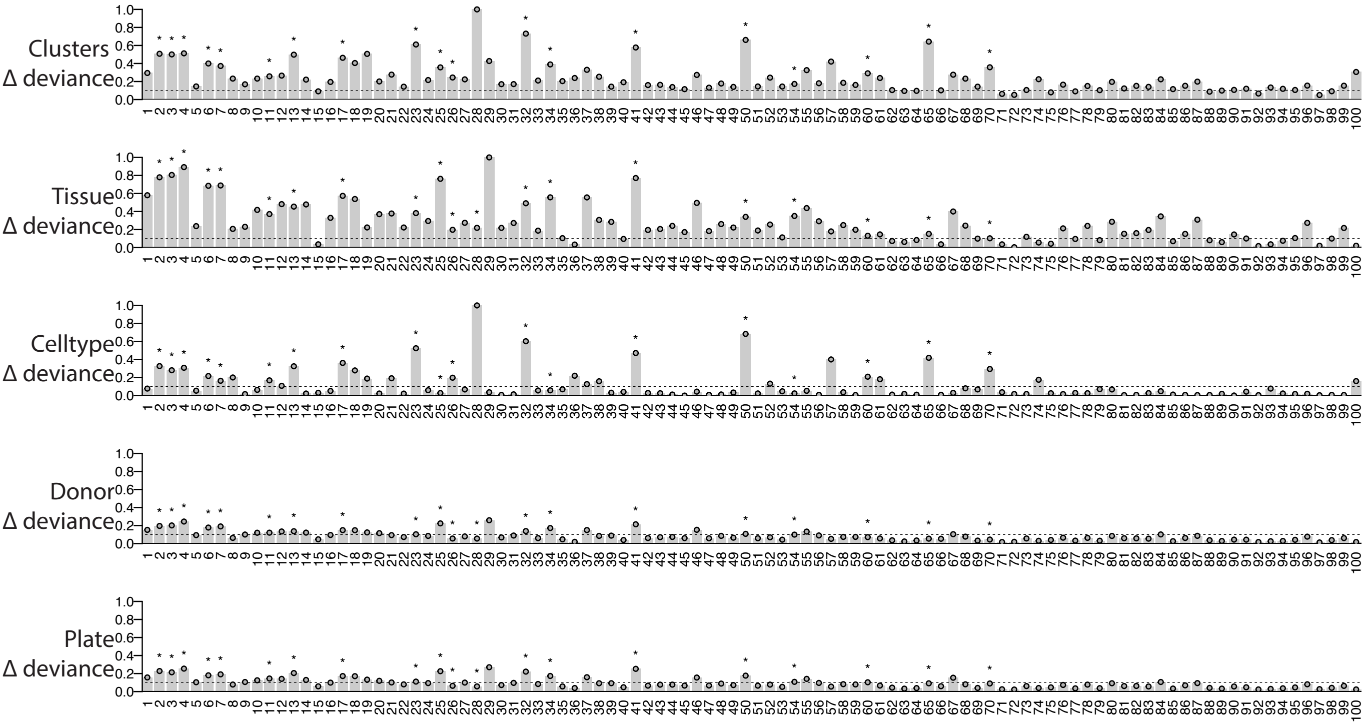

Figure S3. Confusion matrix and delta deviance scores

a) Matrix showing the agreement between unbiased graph-based clustering (columns) and cell surface phenotype as inferred by FACS indexed data (rows). The discordance between cluster and FACS phenotype is shown as a percentage in the bottom row. The % discordance is calculated by dividing the number of cells deviating from the main FACS ILC celltype, by the number of total cells in the cluster, and multiplying the result by 100.

b) Matrix showing the number of cells per donor in each cluster.

c) Bar graph showing the number of cells per donor in each cluster.

d) Bar graph showing the contribution (delta deviance) of each module (1-100) to discriminating clusters, tissues, cell types, donors and plates respectively. Modules described in text are marked with an asterisk.

Data is from 10 independent experiments with one tissue donor each (blood=3, lung=4 and colon=3) integrated with data from [12].
