# Supplementary material for: Tissue-specific transcriptional imprinting and heterogeneity in human innate lymphoid cells revealed by full-length single-cell RNA-sequencing
Source: Cell Res. 2021 Jan 8;31(5):554–68. doi: 10.1038/s41422-020-00445-x (PMC8089104; doi:10.1038/s41422-020-00445-x)
Supplement: Supplementary file 5 — Supplementary Figure S4 [file 41422_2020_445_MOESM5_ESM.pdf]

Figure S4

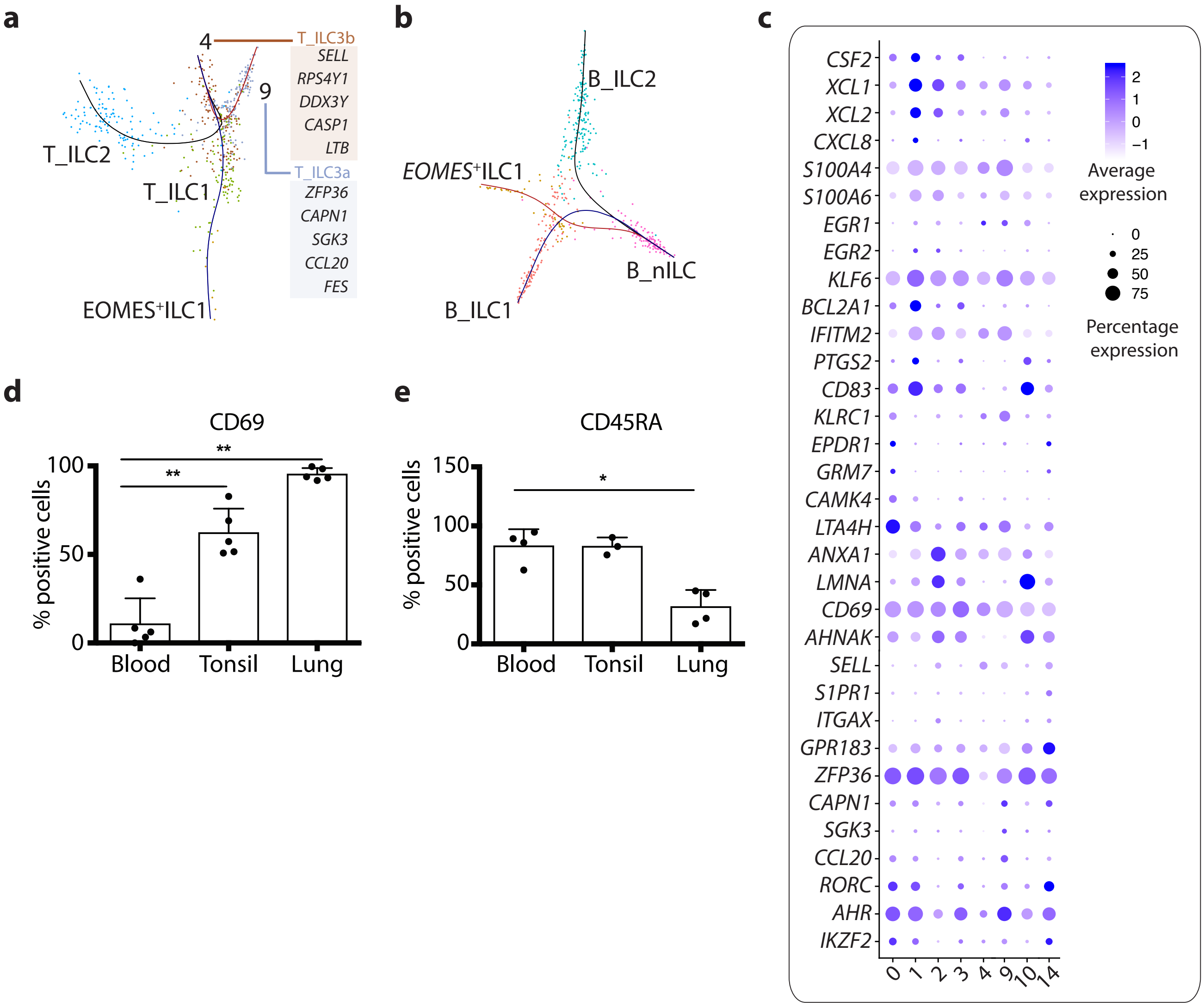

a
4
T_ILC3b
SELL
RPS4Y1
DDX3Y
CASP1
LTB
9
T_ILC3a
ZFP36
CAPN1
SGK3
CCL20
FES
T_ILC2
T_ILC1
EOMES+ILC1
b
B_ILC2
EOMES+ILC1
B_nILC
B_ILC1
c
CSF2
XCL1
XCL2
CXCL8
S100A4
S100A6
EGR1
EGR2
KLF6
BCL2A1
IFITM2
PTGS2
CD83
KLRC1
EPDR1
GRM7
CAMK4
LTA4H
ANXA1
LMNA
CD69
AHNAK
SELL
S1PR1
ITGAX
GPR183
ZFP36
CAPN1
SGK3
CCL20
RORC
AHR
IKZF2
0 1 2 3 4 9 10 14
2
1
0
−1
Average expression
0
25
50
75
Percentage expression
d
CD69
% positive cells
100
50
0
**
**
Blood
Tonsil
Lung
e
CD45RA
% positive cells
150
100
50
0
*
Blood
Tonsil
Lung

Figure S4. Slingshot trajectory analysis of tonsil and *ex vivo* isolated blood, tonsil and lung ILC2 CD69 and CD45RA protein expression.

(**a**-**b**) Slingshot trajectory analysis of tonsil (**a**) and blood (**b**) ILCs displaying a selection of DE genes for each tonsil ILC3 cluster (T_ILC3a-b).

(**c**) Dotplot showing the expression of the top 20 DE genes per ILC3 subcluster in colon, lung and tonsil.

Data is from 10 independent experiments with one tissue donor each (blood=3, lung=4 and colon=3) integrated with data from [12].

(**d**-**e**) Expression of CD69 and CD45RA protein on *ex vivo* isolated blood, tonsil and lung ILC2. ILC2 were selected in a singlet-lymphocyte gate on the basis of FSC/SSC and subsequently gated as follows: DCM (dead cell marker)$^{-}$CD45^{+}lineage (CD1a, CD14, CD19, CD34, CD94, CD123, BDCA2, FcεR1, TCR$\alpha\beta$, TCR$\gamma\delta$)$^{-}$CD3^{-} CD127^{+}CD161^{+}CRTH2^{+}.

Data is from 3-5 independent experiments with 3 tissue donors each (CD69, n=5 for all tissues; CD45RA, blood and lung=4, tonsil=3). Bars in (**d**) and (**e**) represent mean values ± SD. Mann Whitney test *p≤ 0.05, **p≤ 0.01.
