# Supplementary material for: Tissue-specific transcriptional imprinting and heterogeneity in human innate lymphoid cells revealed by full-length single-cell RNA-sequencing
Source: Cell Res. 2021 Jan 8;31(5):554–68. doi: 10.1038/s41422-020-00445-x (PMC8089104; doi:10.1038/s41422-020-00445-x)
Supplement: Supplementary file 6 — Supplementary Figure S5 [file 41422_2020_445_MOESM6_ESM.pdf]

Figure S5

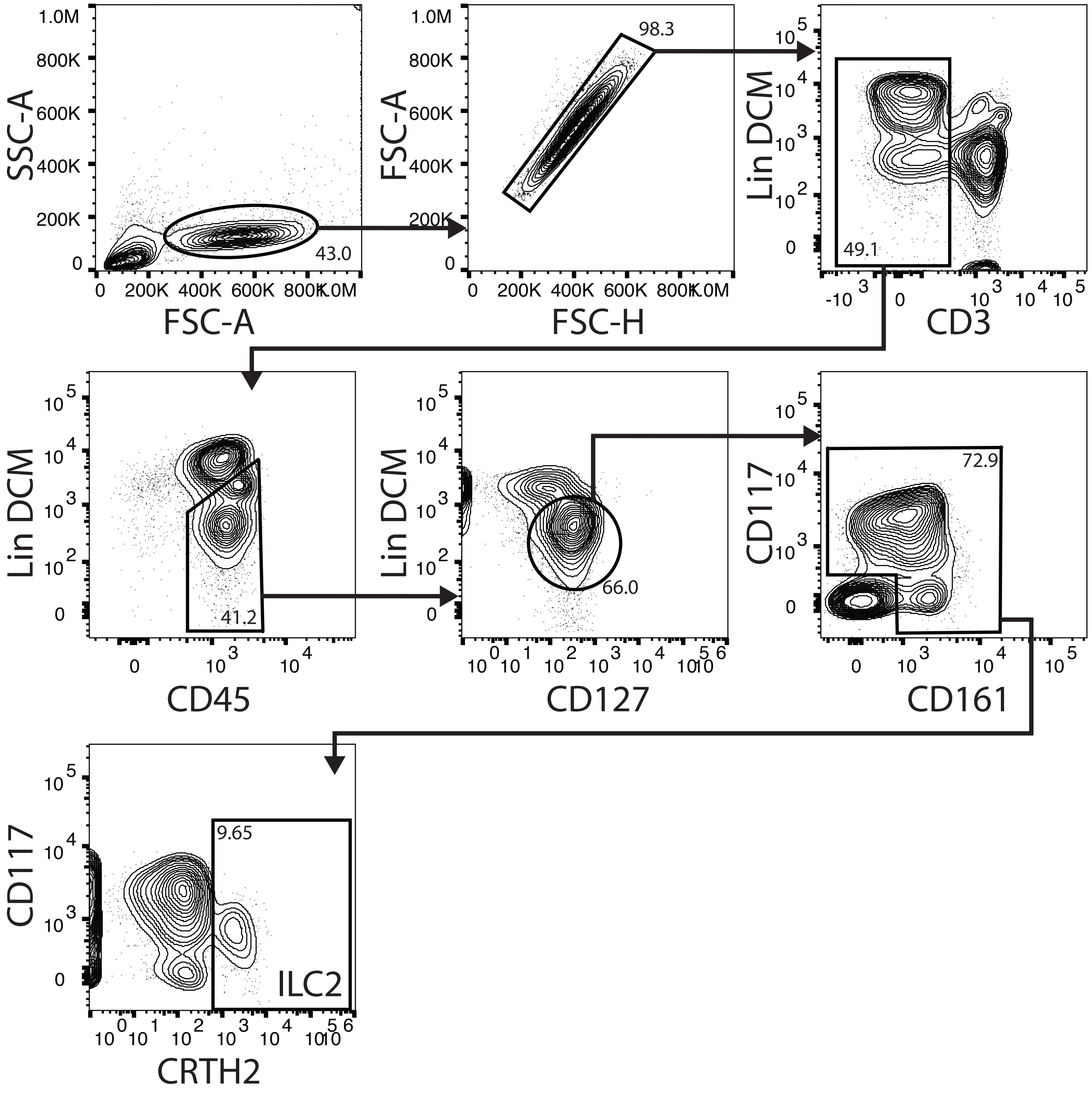
SSC-A
FSC-A
43.0
FSC-H
98.3
Lin DCM
CD3
49.1
CD45
41.2
CD127
66.0
CD117
CD161
72.9
CRTH2
9.65
ILC2

Figure S5. Gating strategy for sorting of blood ILC2

ILC2 were FACS sorted in a singlet-lymphocyte gate on the basis of FSC/SSC and subsequently gated as follows: DCM (dead cell marker)$^{-}$CD45^{+}lineage (CD1a, CD14, CD19, CD34, CD94, CD123, BDCA2, FcεR1, TCR$\alpha\beta$, TCR$\gamma\delta$)$^{-}$CD3^{-}CD127^{+}CD161^{+}CRTH2^{+}. Data is from 7 independent experiments with a total of 13 blood donors
