# Supplementary material for: Tissue-specific transcriptional imprinting and heterogeneity in human innate lymphoid cells revealed by full-length single-cell RNA-sequencing
Source: Cell Res. 2021 Jan 8;31(5):554–68. doi: 10.1038/s41422-020-00445-x (PMC8089104; doi:10.1038/s41422-020-00445-x)
Supplement: Supplementary file 7 — Supplementary Figure S6 [file 41422_2020_445_MOESM7_ESM.pdf]

**Figure S6**

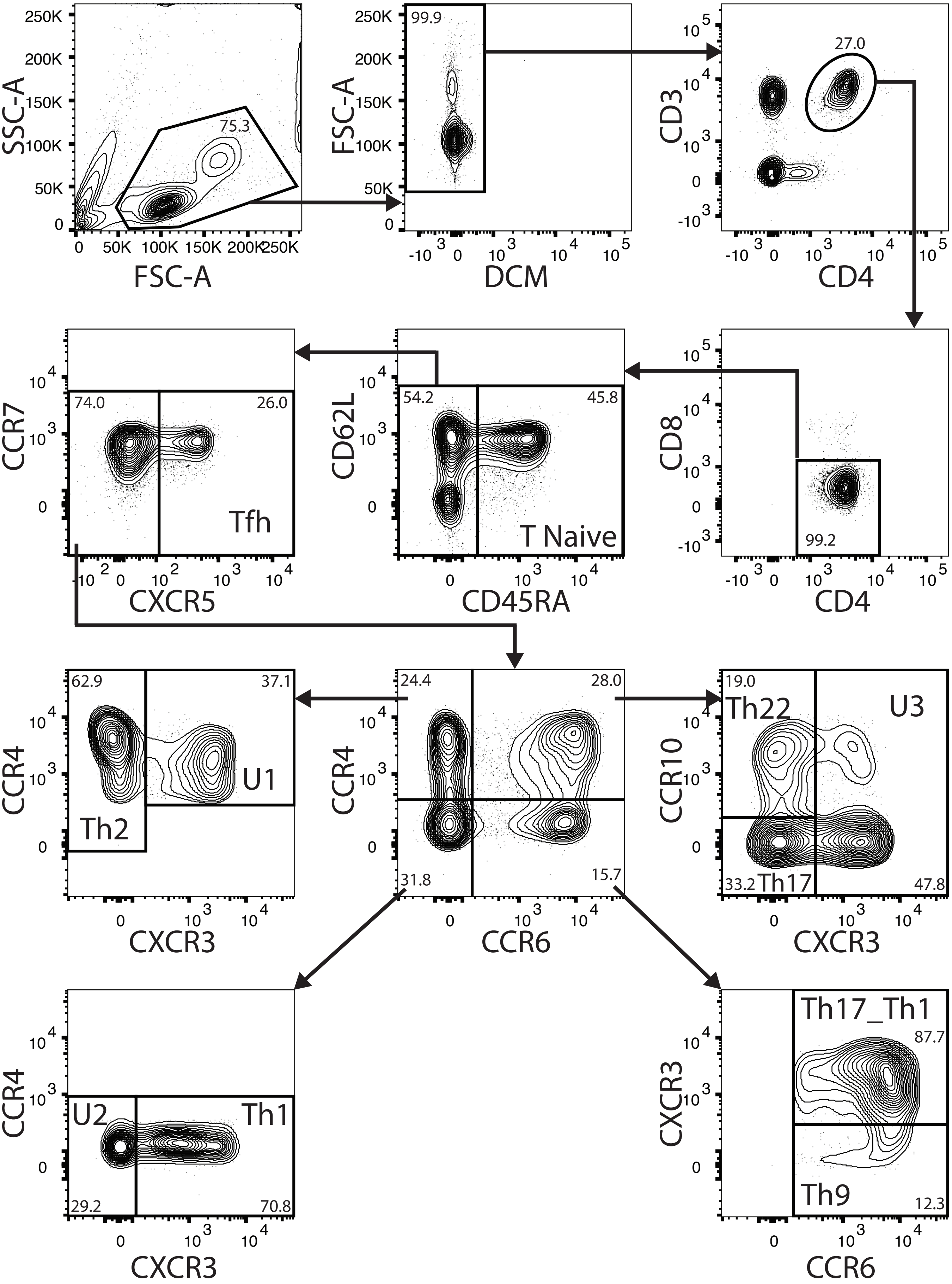

SSC-A
FSC-A
75.3
99.9
DCM
CD3
CD4
27.0
CD8
99.2
CD62L
CD45RA
54.2
45.8
T Naive
CCR7
CXCR5
74.0
26.0
Tfh
CCR4
CCR6
24.4
28.0
31.8
15.7
CXCR3
62.9
37.1
Th2
U1
CCR10
19.0
Th22
U3
33.2
Th17
47.8
U2
Th1
29.2
70.8
Th17_Th1
87.7
Th9
12.3

**Figure S6. Gating for T helper cell subsets (indexed data)**

$CD4^+$ T cells were FACS sorted in a singlet-lymphocyte gate on the basis of FSC/SSC and defined as $CD3^+CD4^+CD8^-$. Additionally, FACS indexed data of chemokine receptor expression was used to define T follicular helper cells (Tfh), Th1, Th17_Th1, Th2, Th9, Th17, Th22, naïve T cells (Tn) and three subsets of undefined T cells (U1-3). Data is from three independent experiments with one blood donor each.
