# Supplementary material for: Tissue-specific transcriptional imprinting and heterogeneity in human innate lymphoid cells revealed by full-length single-cell RNA-sequencing
Source: Cell Res. 2021 Jan 8;31(5):554–68. doi: 10.1038/s41422-020-00445-x (PMC8089104; doi:10.1038/s41422-020-00445-x)
Supplement: Supplementary file 8 — Supplementary Figure S7 [file 41422_2020_445_MOESM8_ESM.pdf]

Figure S7

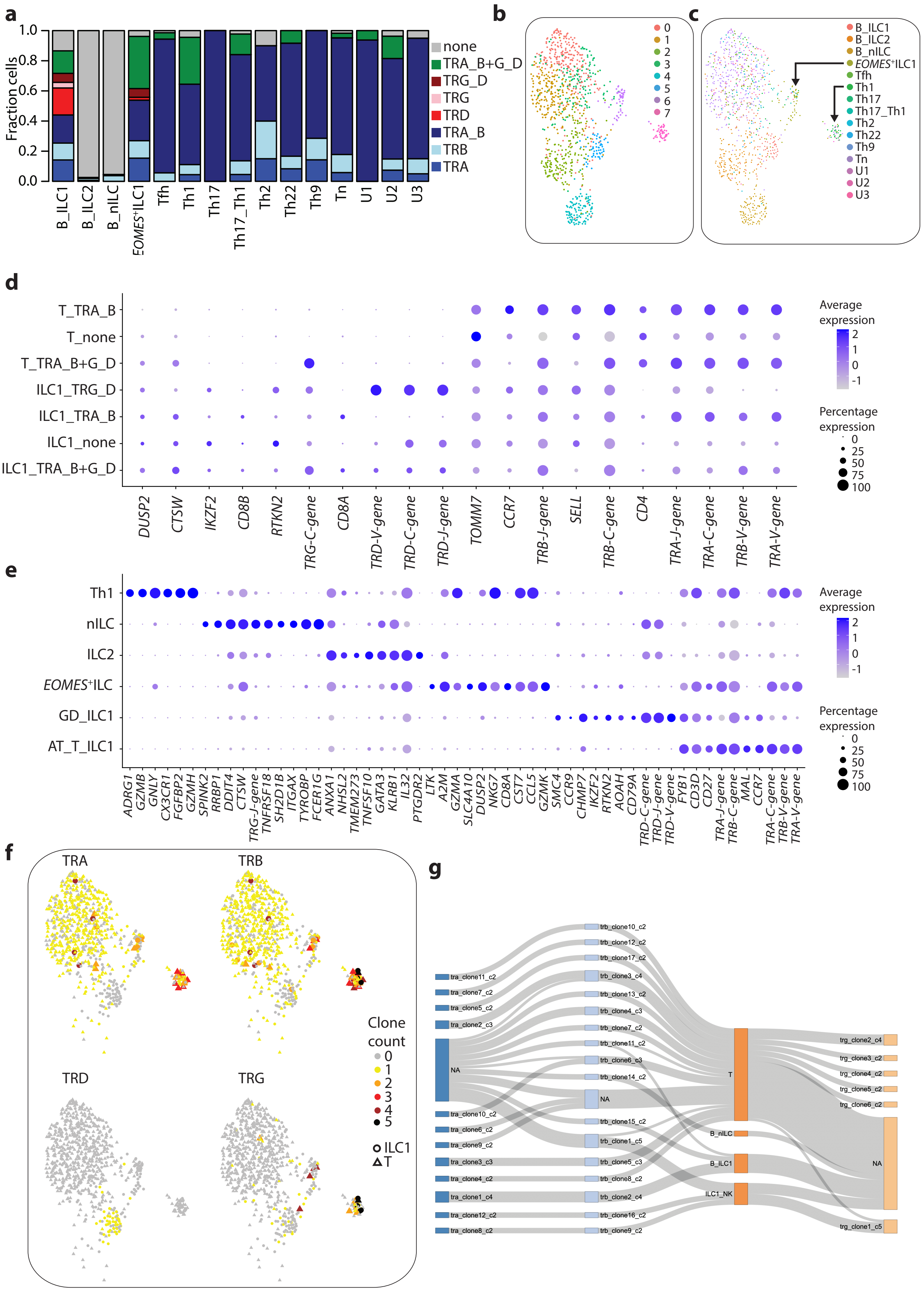
a
Fraction cells
none
TRA_B+G_D
TRG_D
TRG
TRD
TRA_B
TRB
TRA
B_ILC1
B_ILC2
B_nILC
EOMES+ILC1
Tfh
Th1
Th17
Th17_Th1
Th2
Th22
Th9
Tn
U1
U2
U3
b
c
B_ILC1
B_ILC2
B_nILC
EOMES+ILC1
Tfh
Th1
Th17
Th17_Th1
Th2
Th22
Th9
Tn
U1
U2
U3
d
T_TRA_B
T_none
T_TRA_B+G_D
ILC1_TRG_D
ILC1_TRA_B
ILC1_none
ILC1_TRA_B+G_D
DUSP2
CTSW
IKZF2
CD8B
RTKN2
TRG-C-gene
CD8A
TRD-V-gene
TRD-C-gene
TRD-J-gene
TOMM7
CCR7
TRB-J-gene
SELL
TRB-C-gene
CD4
TRA-J-gene
TRA-C-gene
TRB-V-gene
TRA-V-gene
Average expression
Percentage expression
e
Th1
nILC
ILC2
EOMES+ILC
GD_ILC1
AT_T_ILC1
ADRG1
GZMB
GNLY
CX3CR1
FGFBP2
GZMH
SPINK2
RRBP1
DDIT4
CTSW
TRG-J-gene
TNFRSF18
SH2D1B
ITGAX
TYROBP
FCER1G
ANXA1
NHSL2
TMEM273
TNFSF10
GATA3
KLRB1
IL32
PTGDR2
LTK
A2M
GZMA
SLC4A10
DUSP2
NKG7
CD8A
CST7
CCL5
GZMK
SMC4
CCR9
CHMP7
IKZF2
RTKN2
AOAH
CD79A
TRD-C-gene
TRD-J-gene
TRD-V-gene
FYB1
CD3D
CD27
TRA-J-gene
TRB-C-gene
MAL
CCR7
TRA-C-gene
TRB-V-gene
TRA-V-gene
f
TRA
TRB
TRD
TRG
Clone count
ILC1
T
g
NA
T
B_nILC
B_ILC1
ILC1_NK

Figure S7. TCR V(D)J rearrangement analysis of blood ILCs and T cells

a) Predicted V(D)J-rearrangement of *TRA*, *TRB*, *TRD* and/or *TRG* genes in annotated blood ILC clusters (B_ILC1, EOMES$^+$ILC1, B_ILC2 and B_nILC in **Figure 1e**) and in donor matched CD4$^+$ T cell subsets gated according to Figure S6 (Tfh: T follicular helper cells, Tn: naïve T cells, U1-3: unidentified 1-3. TRA_B: rearrangement of *TRA* and *TRB* genes, TRG_D: rearrangement of *TRG* and *TRD* genes, TRA_B+G_D: rearrangement of *TRA* and/or *TCRB* in combination with *TRD* and/or *TRG* genes.

b) UMAP visualization of all blood ILCs and CD4$^+$ T cells color-coded on the basis of unbiased graph-based clustering annotation.

c) UMAP visualization of blood ILCs and CD4$^+$ T cells color-coded on the basis of T helper subset annotation as inferred from FACS indexed data in **Figure S6**.

d) Dotplot showing the expression of the top 10 DE genes between ILC1 (cells annotated as B_ILC1 merged with *EOMES*$^+$ILC1 in **Figure 1e**) and T cells as inferred from FACS indexed data. Gene expression is displayed separately depending on TCR (V(D)J rearrangement pattern. TRA_B: rearrangement of *TRA* and/or *TRB* genes, TRG_D: rearrangement of *TRG* and/or *TRD* genes, TRA_B+G_D: rearrangement of *TRA* and/or *TCRB* in combination with *TRD* and/or *TRG* genes.

e) Dotplot showing the expression of the top DE genes for each cluster as annotated in **Figure 7d** (Th1, nILC, ILC2, AB_T_ILC1, GD_ILC1 and *EOMES*$^+$ ILC1 (bottom).

f) UMAP visualization of blood ILCs (cells annotated as B_ILC1 merged with *EOMES*$^+$ILC1 in **Figure 1e**) and CD4$^+$ T cells (annotated from FACS indexed data, Figure S6). Cells are color-coded on the basis of CDR3-region clone count and shape-coded by cell type.

g) Sankey diagram showing the overlap of CDR3 clone identity for TRA, TRB, TRG and celltype (ILCs annotated by clustering in Figure 1e and T cells on FACS phenotype inferred from indexed data). Only cells with clone count >= 2 for any rearranged chain is included.

Data is from three independent scRNAseq experiments with one blood donor each.
