# Supplementary material for: Tissue-specific transcriptional imprinting and heterogeneity in human innate lymphoid cells revealed by full-length single-cell RNA-sequencing
Source: Cell Res. 2021 Jan 8;31(5):554–68. doi: 10.1038/s41422-020-00445-x (PMC8089104; doi:10.1038/s41422-020-00445-x)
Supplement: Supplementary file 9 — Supplementary Figure S8 [file 41422_2020_445_MOESM9_ESM.pdf]

Figure S8

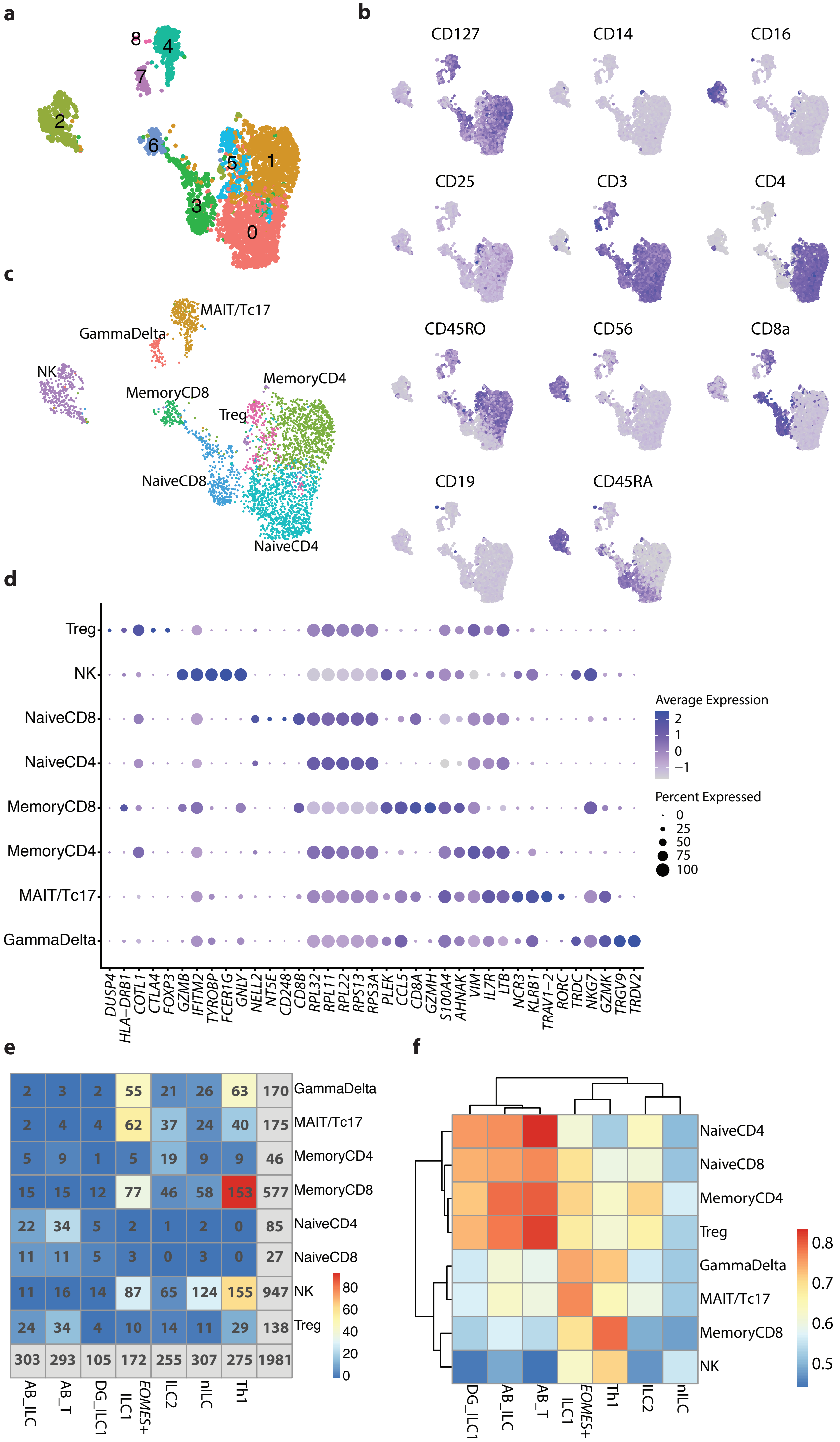

a
8
4
7
2
6
5
1
3
0
b
CD127
CD14
CD16
CD25
CD3
CD4
CD45RO
CD56
CD8a
CD19
CD45RA
c
MAIT/Tc17
GammaDelta
NK
MemoryCD4
MemoryCD8
Treg
NaiveCD8
NaiveCD4
d
Treg
NK
NaiveCD8
NaiveCD4
MemoryCD8
MemoryCD4
MAIT/Tc17
GammaDelta
DUSP4 HLA−DRB1 COTL1 CTLA4 FOXP3 GZMB IFITM2 TYROBP FCER1G GNLY NELL2 NT5E CD248 CD8B RPL32 RPL11 RPL22 RPS13 RPS3A PLEK CCL5 CD8A GZMH S100A4 AHNAK VIM IL7R LTB NCR3 KLRB1 TRAV1−2 RORC TRDC NKG7 GZMK TRGV9 TRDV2
Average Expression
2
1
0
−1
Percent Expressed
0
25
50
75
100
e
2 3 2 55 21 26 63 170 GammaDelta
2 4 4 62 37 24 40 175 MAIT/Tc17
5 9 1 5 19 9 9 46 MemoryCD4
15 15 12 77 46 58 153 577 MemoryCD8
22 34 5 2 1 2 0 85 NaiveCD4
11 11 5 3 0 3 0 27 NaiveCD8
11 16 14 87 65 124 155 947 NK
24 34 4 10 14 11 29 138 Treg
303 293 105 172 255 307 275 1981
AB_ILC
AB_T
DG_ILC1
ILC1 EOMES+
ILC2
nILC
Th1
80
60
40
20
0
f
NaiveCD4
NaiveCD8
MemoryCD4
Treg
GammaDelta
MAIT/Tc17
MemoryCD8
NK
DG_ILC1
AB_ILC
AB_T
ILC1 EOMES+
Th1
ILC2
nILC
0.8
0.7
0.6
0.5

Figure S8. 10x data integration

a) UMAP with clustering of 10x PBMC data (pre-selected $CD3^+$ and $CD56^+CD3^-$ cells) with 8 clusters.

b) UMAPs with cell surface protein detection with antibody derived tags (ADT)

c) UMAP with cluster 8 removed and annotated by celltype.

d) Dotplot with top DEGs for each of the clusters.

e) Differentially expressed genes (DEGs) overlap for blood SS2 (x-axis) and 10x PBMC (y-axis) data. All DEGs between clusters within each of the datasets were run separately. The numbers in gray boxes at bottom/right part is the number of DEGs (only upregulated genes) per cluster and numbers in coloured boxes is the common genes to two lists. Colors of the boxes are -log10(p-value) from phyper test for significance of overlap. Cells in the AB_T_ILC1 cluster were separated into T-cells (AB_T) and ILCs (AB_ILC) for this analysis.

f) Heatmap with pairwise spearman correlation between clusters in the SS2 data (x-axis) and the 10x PBMC data (y-axis). Mean expression per cluster was calculated using the union of all top 100 upregulated DEGs per cluster from both datasets and used to calculate the correlations.
